# Disordered DNA methylation leads to targetable transcriptional plasticity in ATRT

**DOI:** 10.1186/s40478-025-02173-y

**Published:** 2025-12-17

**Authors:** Ashley R. Tetens, Tyler R. Findlay, Jordyn Craig-Schwartz, Athanasia Liapodimitri, Oscar Camacho, Kegan O. Skalitzky, Adrian Idrizi, Rakel Tryggvadottir, Kayleigh Lunsford, Eric H. Raabe, Michael A. Koldobskiy

**Affiliations:** 1 Center for Epigenetics, Johns Hopkins University School of Medicine, Baltimore, MD 21205 USA; 2 Pediatric Oncology, Sidney Kimmel Comprehensive Cancer Center, Johns Hopkins University School of Medicine, Baltimore, MD 21231 USA

## Abstract

Atypical teratoid rhabdoid tumor (ATRT) is a highly aggressive but genetically simple pediatric central nervous system tumor, defined by biallelic inactivation of the chromatin regulator *SMARCB1* with remarkably few other cooperating mutations. Despite its genetic homogeneity, ATRT exhibits profound clinical and epigenetic heterogeneity, with three major subgroups (ATRT-TYR, ATRT-MYC, and ATRT-SHH) defined by DNA methylation and transcriptional signatures. Beyond these subgroup-defining features, we aimed to investigate epigenetic variability within tumors by applying whole-genome bisulfite sequencing and probabilistic modeling to quantify stochastic DNA methylation in primary ATRT samples encompassing all three subgroups. We show that ATRT exhibits a destabilized and increasingly stochastic methylome. While ATRT global methylation patterns diverge according to subgroup, some methylation perturbations, such as hypermethylation and increased methylation entropy over bivalent promoters, are consistent across subgroups. We find that methylation stochasticity alterations map onto potential drivers of ATRT, such as *LIN28a*, the *HOXD* cluster for ATRT-MYC, and *OTX2* for ATRT-TYR, and identify actionable targets, such as hypermethylation of the tumor suppressor *CDKN2a* across all subgroups. We investigate the sensitivity of the aberrant DNA methylation landscape of ATRT to pharmacologic DNA methyltransferase inhibition (DNMTi) and histone deacetylase inhibition (HDACi). We show that decitabine leads to profound demethylation of patient-derived ATRT cell lines, including reversal of hypermethylation at bivalent promoters and the *CDKN2a* locus. The addition of HDACi leads to dramatic gene expression changes, including upregulation of innate immune signaling pathways, such as STING/interferon signaling, genes under the regulation of bivalent promoters, and reactivation of the tumor suppressor CDKN2A. The combination of DNMTi and HDACi synergistically reduces cell viability. Taken together, we show that ATRT has a highly stochastic methylome sensitive to epigenetic manipulation.

**Supplementary Information:**

The online version contains supplementary material available at 10.1186/s40478-025-02173-y.

## Introduction

Atypical Teratoid Rhabdoid Tumor (ATRT) is an embryonal CNS tumor of early childhood with a poor prognosis, characterized by biallelic inactivation of *SMARCB1* (or, less commonly, *SMARCA4)*, a core component of the SWI/SNF chromatin remodeling complex [12, 50]. Remarkably, the mutational burden in ATRT is exceptionally low, among the lowest of any cancer [33]. Despite its simple genome, ATRT represents a heterogenous clinical entity with divergent anatomic location, age of onset, and treatment outcomes, leading to the recognition of 3 distinct ATRT subgroups: ATRT-MYC, ATRT-TYR, and ATRT-SHH. The subgroups were categorized according to differences in gene expression and epigenetic features, including DNA methylation patterns and enhancer networks, suggesting that the observed clinical heterogeneity of ATRT was underscored by divergent epigenetic landscapes [27, 48]. There has been intense interest in defining the targets of epigenetic dysregulation downstream of SMARCB1 loss [37]. Normally, the SWI/SNF complex restructures repressive chromatin and is responsible for the recruitment of histone acetyltransferases (HATs) to their enhancers. SMARCB1 mutation is linked to a reduction in histone acetylation (specifically H3K27ac), particularly at enhancers associated with differentiation. Additionally, regions of the genome repressed by PRC2-mediated deposition of H3K27me3 in normal tissue become disrupted in ATRT, leading to reduced H3K27me3 with a concomitant increase in DNA methylation in these regions [15]. These epigenetic alterations may lead to a block on normal lineage specification and cell fate determination, thus promoting tumorigenesis.

Though DNA methylation has previously been studied in ATRT, it has not been investigated in a way that evaluates methylation variability. Prior studies have relied on either methylation arrays which do not capture the full methylome or have analyzed whole genome bisulfite sequencing (WGBS) data using approaches that smooth methylation data and do not evaluate methylation variability [27, 48]. Epigenetic variability is increasingly appreciated as a driving force of intratumoral heterogeneity, phenotypic plasticity, and therapeutic resistance, and increased stochastic variation of DNA methylation correlates with gene expression variability at the single-cell level [32, 47]. It is thus imperative to capture the methylation variability of ATRT, as it could point to the molecular underpinnings of ATRT transformation and therapeutic resistance. To this end, we applied a recently developed approach that employs principles from statistical physics and information theory to derive DNA methylation potential energy landscapes from WGBS data, allowing the quantification of DNA methylation stochasticity using the information-theoretic measure of normalized Shannon entropy. We and others have shown that this method can identify the targets of epigenetic instability in cancer, including the identification of non-mutated, epigenetically altered driver genes in acute lymphoblastic leukemia [30].

We performed WGBS on 22 primary ATRT samples encompassing all three disease subgroups, along with 4 normal fetal brain controls, and subjected these to an analysis which models DNA methylation potential energy landscapes and quantifies methylation stochasticity genome-wide [29, 46]. Our results reveal subgroup-specific differences in methylation entropy, with ATRT-MYC exhibiting the highest intratumoral heterogeneity. Gene targets of differential methylation stochasticity are enriched in developmental and chromatin regulatory genes, implicating stochastic methylation in epigenetic plasticity and subgroup identity. Comparison with previously published transcriptomic data suggests that stochastic methylation contributes to dysregulated gene expression programs in a context-dependent manner. These findings can advance our understanding of epigenetic heterogeneity in ATRT and highlight potential methylation-driven mechanisms underlying subgroup-specific biology and therapeutic vulnerability.

Interestingly, although the global patterns of mean methylation and methylation entropy diverged according to subgroup, we also identify recurrent targets of altered methylation stochasticity, including putative epigenetic drivers of ATRT, across all ATRT subgroups. Given our finding that ATRT has a markedly disordered methylation landscape, we hypothesized that it would be sensitive to hypomethylation by the DNA methyltransferase inhibitor (DNMTi), decitabine. Additionally, prior studies demonstrated that *SMARCB1* inactivation leads to low levels of H3K27ac globally and at enhancers related to lineage specification. The combination of DNMTi and HDACi has been shown to reduce cell viability and modulate immunogenicity in a variety of cancer types via induction of endogenous retroviral elements and neoantigens [5, 21, 46]. Further, the combination of DNMTi and HDACi has been shown to have mechanistic synergy, whereby DNA hypomethylation enhanced HDACi-mediated hyperacetylation and transcriptional response [2]. We thus sought to examine the effect of combination treatment with decitabine and the HDAC inhibitor, RG2833, in ATRT. We show that the combination of these two agents in patient-derived ATRT cell lines leads to dramatic changes in gene expression, including activation of innate immune signaling via the STING/interferon pathways, upregulation of genes controlled by bivalent promoters or SMARCB1 expression, and re-expression of silenced tumor suppressors such as *CDKN2A.* This is accompanied by a synergistic reduction in cell viability. Taken together, this study maps the stochastic methylome of ATRT and shows that targeting the aberrant epigenetic landscape of ATRT leads to transcriptional remodeling that can be exploited therapeutically.

## Materials and methods

### Patient primary samples

Primary patient samples were obtained from the Children's Brain Tumor Network (CBTN) at the Children’s Hospital of Philadelphia (CHOP). Full description of samples is provided in Table S1.

### WGBS library preparation, sequencing, and analysis

For primary patient ATRT cell samples, we isolated genomic DNA and prepared samples for sequencing following methods previously described [29]. Alignment, genomic feature identification and annotations were performed as described previously [29]. Methods for computation of DNA methylation potential energy landscapes (PELs) from WGBS data are previously described and performed using the informME (v0.3.2) analysis pipeline [29].

For methylation-based classification of brain tumor samples, BAM files were processed using the methylation_extractor function from Bismark to generate bedMethyl files, trimming the ends of the reads showing methylation bias. The resulting data were analyzed with the MNP-Flex platform (v12.8) for tumor classification [41].

After dividing the genome into non-overlapping genomic windows of 3 kilobases (kb) for PEL construction, we further partitioned each 3-kb estimation window into 20 non-overlapping analysis regions of 150 base pairs. We then used the PELs to generate methylation probability distributions using the Mean Methylation Level (MML) and Normalized Methylation Entropy (NME). We additionally performed differential methylation analysis between a test (ATRT) and reference (normal fetal brain) to generate differential MML (dMML) and differential NME (dNME). We computed the Jensen-Shannon distance (JSD) between two probability distributions of the methylation level in a test (ATRT) and a reference (normal) sample region using previously described methods [29]. For evaluation of DNA methylation changes over previously annotated chromatin classes, we used chromHMM annotations for E0053, cortex-derived primary neurospheres, as a reference cell type [16]. To identify high JSD regions that are predominantly driven by dNME, rather than dMML, we used a relative JSD ranking approach (rJSD) as described previously [26].

### Cell culture

All ATRT cell lines (CHLA02, CHLA05, CHLA06, and CHLA266) were provided by Dr. Jeffrey Rubens of The Johns Hopkins Hospital. Cell lines were cultured in DMEM/F12 human neurosphere proliferation medium (30% Hams F12 [Gibco 11765070], 70% DMEM [Gibco 11995073], 1% Antibiotic–Antimycotic [15240062], 2% B27 minus Vitamin A [12587001], 20 ng/ml EGF [Peprotech AF-100-15], 20 ng/ml FGF-2 [Peprotech 100-18B], and 5 µg/ml of heparin [Millipore Sigma H3149]. All cell lines were kept in 25 cm^2^ culture flasks at 37 °C and 5% CO_2_. The hypomethylating agent, 5-aza-2’-deoxycitine (Decitabine; Cayman Chemical; #11166), and the HDACi, RG2833 (Adooq Bioscience; #A13139), were used in our cell treatments. All cell culture experiments were performed using a 5-day pretreatment with 100 nM DAC or vehicle. This treatment included daily media changes. 5 µM RG2833 was added on day 5 and cells were harvested for subsequent analysis at the 72-h time point, unless specified otherwise.

### RNA sequencing and analysis

Strand specific mRNA libraries were generated by Novogene using the NEBNext Ultra II Directional RNA library prep Kit for Illumina (New England BioLabs #E7760), mRNA was isolated using Poly(A) mRNA magnetic isolation module (New England BioLabs #E7490). Stranded mRNA libraries were sequenced by Novogene on an Illumina NovaSeq6000 instrument, using 150 bp paired-end dual indexed reads. All analyses were done using a threshold of > 50.0 base mean and a Log_2_ fold change > 1.50, unless otherwise specified.

### Protein analysis

Protein Analysis was performed by western blotting with methods previously described [46]. Protein levels were assessed using the following antibodies: IFITM3 (1:1000; Cell Signaling Technology; #59212S), IRF-7 (1:200; Cell Signaling Technology #13014), GAPDH control (1:10,000; Santa Cruz; #SC-47724), STING (1:200; Cell Signaling Technology, #13647S), alpha-tubulin (1:1,000; #2144S) and p16 INK4A (1:100, Cell Signaling Technology #80772S).

### Bliss synergy experiment

ATRT cell lines (CHLA02, CHLA05, and CHLA06) were pre-treated with decitabine (DAC) in flasks for 5 days at either 0, 25, 50, 75, or 100 nM, with daily media changes. On day 5, pre-treated cells were moved to 6-well plates at 5.0e4 cells per mL with each concentration of DAC being treated with a single dose of RG2833 (0, 0.5, 1.0, 1.5, 2.0 µM). Cell viability was assessed using Muse Count & Viability Kit (Cytek Biosciences, Cat. No. MCH100102; Fremont, CA, USA) according to manufacturer’s protocol. Viability was assessed 5 days after single RG2833 treatment for CHLA02/05, and 3 days after for CHLA06, due to differences in growth rates. Synergy score analysis was calculated based on Bliss independence model through SynergyFinder v3.0 web application (https://synergyfinder.fimm.fi/) [23].

## Results

### DNA methylation potential energy landscape analysis identifies increased DNA methylation entropy in ATRT-MYC and ATRT-SHH subgroups

WGBS was performed on 22 primary ATRT samples and subjected to potential energy landscape (PEL) analysis using the informME pipeline (sample characteristics described in Supplementary Table 1). InformME applies principles from the Ising Model of statistical physics to generate PELs of methylation based on the probability distribution of methylation levels throughout the genome, encapsulating local variation in DNA methylation patterns [29]. To relate DNA methylation to ATRT molecular subgroups, samples were classified based on WGBS data using the MNP-flex methylation classifier (Supplementary Table 2) [41]. Samples with clustering score > 0.5 were treated as reliably assigned to a subgroup (16 of 22); previous studies have shown that clustering scores of at least 0.3 based on non-array data have a 99.2% subgroup classification accuracy [41].

**Fig. 1:**
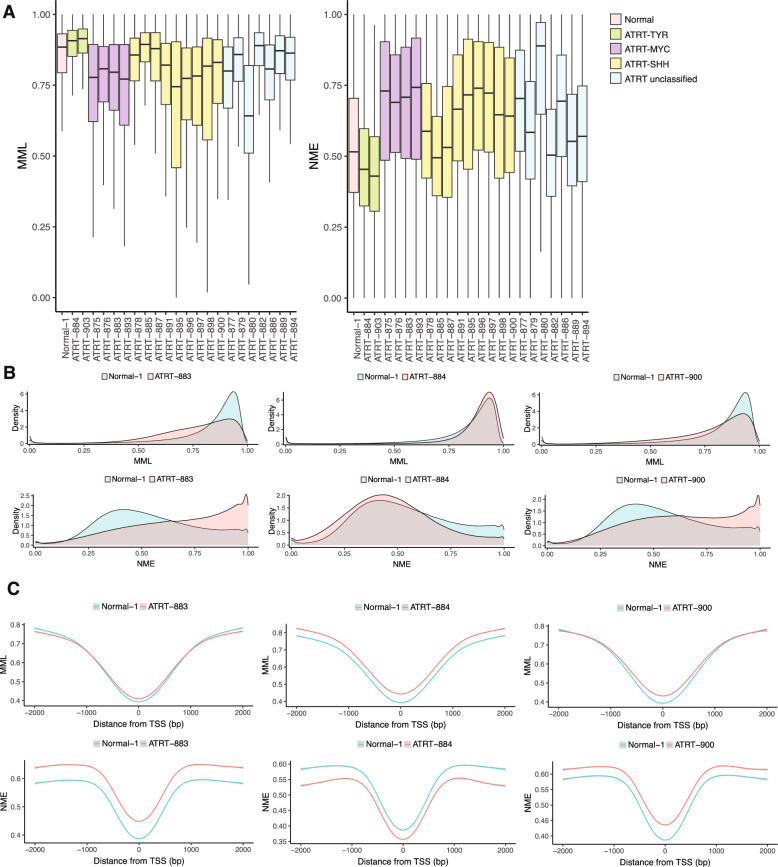
WGBS analysis of ATRT reveals disordered methylation patterns genome-wide that diverge according to subgroups. **A** Box plots of genome-wide mean methylation levels (MML) (left) and normalized methylation entropy (NME) (right) in 22 primary patient samples of clinically diagnosed ATRT and 1 normal fetal brain control. Pink, control; green, ATRT-TYR; purple, ATRT-MYC; yellow, ATRT-SHH; blue, unclassified. Center line represents the median, box is the interquartile range (IQR), and whiskers are 1.5 × IQR. **B** Density distributions of genome-wide MML (top) and NME (bottom) for control (blue) and ATRT (pink). From left to right; ATRT-883, ATRT-884, ATRT-900. **C** Smoothed genome-wide MML (top) or NME (bottom) ± 2 kb of TSSs for control (blue) and test (pink). From left to right: ATRT-883, ATRT-884, and ATRT-900

In agreement with prior reports, we found that ATRT primary patient samples had marked differences in mean methylation levels (MML), largely aligning with subgroup [27]. The mean methylation level (MML) describes the average amount of CpG methylation in a genomic unit, ranging from 0 to 1, with 0 representing a completely unmethylated region and 1 representing a maximally methylated region. Compared to normal fetal brain, the ATRT-TYR group exhibited global hypermethylation, the ATRT-MYC subgroup demonstrated global hypomethylation, and the ATRT-SHH group had variable MML distributions, with a trend towards hypomethylation (Fig. 1a). Previous studies have also noted that ATRT-SHH is a heterogenous subtype and may be subdivided into further clusters but typically trends towards global hypermethylation. However, we find here that most of our ATRT-SHH samples exhibit genome-wide hypomethylation, with only a few samples being hypermethylated [18, 27].

Normalized methylation entropy (NME) quantifies the extent of stochastic variation in a genomic unit using Shannon’s entropy, normalized for the number of CpG’s in a region. NME ranges from 0 to 1, where 0 represents a fully ordered (deterministic) methylation distribution, and 1 represents a maximally disordered (random) distribution. We observed differences in global DNA methylation entropy according to ATRT subgroup, which has not been reported previously (Fig. 1a). While the majority of the ATRT samples in ATRT-MYC and ATRT-SHH groups had significantly elevated NME, the ATRT-TYR group had overall decreased genome-wide methylation entropy, as compared to normal fetal brain reference. Using representative samples with the highest classification score for each ATRT subgroup (ATRT-883 for MYC, ATRT-884 for TYR, and ATRT-900 for SHH), we evaluated genome-wide densities of MML and NME distributions (Fig. 1b). ATRT-MYC sample, ATRT-883, and ATRT-SHH sample, ATRT-900, demonstrate a genome-wide shift toward lower MML and markedly increased NME, including a proportion of the genome that is maximally disordered, with NME near or equal to 1 (Fig. 1b), while ATRT-TYR sample, ATRT-884, shows elevated MML and globally decreased NME. When evaluating the distribution of MML and NME within 2 kb of the transcription start sites (TSS) for all genes, we see similar trends, in addition to slight hypermethylation over the TSS for each sample (Fig. 1c). Overall, this data indicates that ATRT primary patient samples exhibit global perturbations in methylation entropy as well as mean methylation, with both MML and NME identifying subgroup-specific differences.

### Methylation discordance maps onto key genomic regions

**Fig. 2:**
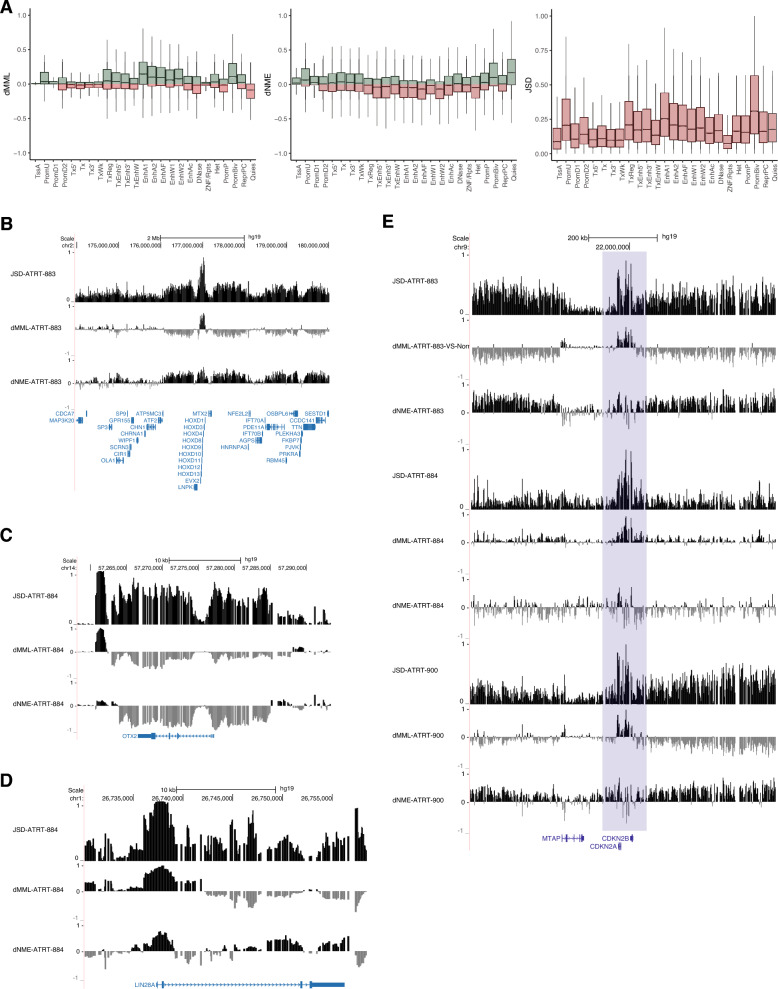
Disordered methylation patterns converge on core genomic structural features and key genes. **A** Boxplots of differential mean methylation levels (dMML) (left), differential normalized methylation entropy (dNME) (center), and Jensen-Shannon Divergence (JSD) (right) for 25 ChromHmm genomic annotations in ATRT-883. Center line is the median, box is the IQR, and whiskers are 1.5 × IQR. **B** Tracks representing JSD (top), dMML (middle), and dNME (bottom) over the HOXD cluster in ATRT-883. **C** Tracks representing JSD (top), dMML (middle), and dNME (bottom) over *OTX2* in ATRT-884. **D** Tracks representing JSD (top), dMML (middle), and dNME (bottom) over *LIN28a* in ATRT-884. **E** Tracks representing JSD, dMML, and dNME over the *CDKN2A* locus in ATRT-883 (top), ATRT-884 (middle), and ATRT-900 (bottom). Blue region highlights the CDKN2A gene and the peak on chromosome 9

We then set out to map targets of differential DNA methylation stochasticity in ATRT, assessing differential MML, differential NME, and the Jensen-Shannon distance (JSD), reflecting the dissimilarity of methylation probability distributions in a genomic region between samples [30]. Evaluating differential methylation stochasticity over genomic features, we found that, in most samples, the greatest discordance in methylation stochasticity (highest JSD) was observed over CpG islands, which also demonstrate hypermethylation even in ATRT-MYC and ATRT-SHH samples that otherwise demonstrate genome-wide hypomethylation (Supplementary Fig. 1a). We then evaluated differential methylation stochasticity across different chromatin classes, as annotated using ChromHMM for normal cortex-derived primary neurospheres, partitioning the genome into distinct regulatory classes including specific promoter and enhancer subtypes, heterochromatin, quiescent chromatin, and others [16]. Boxplots for ATRT-883 (MYC) are shown in Fig. 2a, and ATRT-884 (TYR) and ATRT-900 (SHH) are shown in Supplementary Fig. 1b. In all representative samples, regions that are annotated as bivalent promoters in normal cells demonstrated high JSD values in ATRT, which was driven by mean hypermethylation and dramatically increased methylation entropy. The local distribution of MML and NME around normally bivalent promoters confirms this finding (Supplementary Fig. 2c). Of interest, certain enhancer classes (EnhA1, EnhA2, EnhAF, EnhW1, EnhW2,) are similarly hypermethylated, but have reduced methylation entropy, reflecting a more deterministic methylation state. Other regions with high JSD included transcription regulatory regions (TxReg) and upstream promoters (PromU), with relative hypermethylation of both regions but elevated entropy over PromU and reduced entropy at TxReg. Interestingly, although different subgroups of ATRT had global methylation differences, their trends at specific regulatory regions were remarkably similar. This suggests that, regardless of subtype, methylation stochasticity maps onto key genomic regions and chromatin classes in similar patterns, particularly at bivalent promoters, enhancers, transcription regulatory regions, and upstream promoters, as annotated in the normal reference cell type.

### Genes exhibiting altered methylation stochasticity are enriched in key pathways

Given substantial global and local alterations in DNA methylation stochasticity in ATRT, we aimed to identify genes and gene set enrichments affected by these changes. For each ATRT sample, we ranked genes by JSD over promoter regions, defined as TSS ± 2 kb (Supplementary Table 3a,c,g). To identify subgroup-specific enrichments, we focused on overlaps among genes exhibiting the highest JSD (top 1000) within each subgroup. For ATRT-MYC, we identified 252 genes that consistently exhibit the highest discordance in methylation stochasticity (Supplementary Table 3a). Using the Gene Set Enrichment Analysis (GSEA) tool, we identified top enrichments related to the Polycomb Repressive Complex 2 (PRC2), including targets of EED and SUZ12, as well as genes associated with H3K27me3 [36, 44]. Additionally, there were enrichments of genes related to neurogenesis, organ morphogenesis, embryonic organ development, homophilic cell adhesion, and CNS development (Supplementary Table 3b).

Because only two samples were available within the ATRT-TYR subgroup, these were evaluated both as an overlap set of 263 genes (Supplementary Tables 3c-d) and individually (Supplementary Tables 3e-f). Similar to ATRT-MYC, genes with highest JSD for each sample demonstrated GSEA C2 enrichments for SUZ12 and EED targets, genes under the regulation of H3K27me3 and bivalent promoters, and gene sets related to differentiation (Supplementary Tables 3e-f). For ATRT-SHH, we observed greater sample to sample heterogeneity in methylation features. The overlapping gene list among the top 1000 JSD genes across 9 ATRT-SHH samples contained only 74 genes (Supplementary Table 3 g). Again, among the top 50 C2 and C5 GSEA enrichments were targets of SUZ12 and EED, genes regulated by bivalent promoters (demonstrated by H3K4me3 and H3K27me3 marks), and genes involved in organ morphogenesis, anatomical structure formation, and transcription regulator activity (Supplementary Table 3 h). PRC2 and SMARCB1 play antagonistic roles, such that biallelic inactivation of SMARCB1 may poise PRC2 to modulate chromatin states unchecked. Even with SMARCB1 loss, however, there is residual SWI/SNF activity, which may antagonize PRC2 activity [7, 15, 55]. The enrichment of PRC2-regulated genes among genomic regions with highly stochastic methylation suggests a mechanistic interplay between PRC2 and DNA methylation at these regions.

Interestingly, 11 genes had highly discordant methylation stochasticity in all classified ATRT samples (Supplementary Table 3i), notably including *Gli2. Gli2* has been reported to be highly overexpressed in the ATRT-SHH subgroup and is the target of 2 super-enhancers in this subtype [27]. Here, we show that *Gli2* is the target of methylation stochasticity across all ATRT samples we analyzed, regardless of subtype.

### Enhanced methylation stochasticity maps onto the promoter regions of key genes

To further characterize the role of methylation stochasticity in ATRT, we evaluated local methylation features of genes with the highest JSD in ATRT subgroups (Supplementary Table 3). We find multiple HOX cluster genes (A, B, C, and D), with an emphasis on HOXD, among the top 1000 genes by JSD in multiple ATRT samples, particularly the ATRT-MYC subgroup (Supplementary Table 3a). Using the UCSC genome browser, we show that in ATRT-883, the representative ATRT-MYC sample, there is a striking elevation in JSD over the entire HOXD cluster, driven by focal hypermethylation over HOXD cluster genes and markedly elevated methylation entropy (Fig. 2b) [42].

In the ATRT-TYR subgroup, *OTX2* is identified as a gene with altered methylation stochasticity compared to normal fetal brain control. *OTX2* encodes a neural transcription factor that has been reported to be under the regulation of a super-enhancer in ATRT [27]. Additionally, OTX2 is over-expressed in the ATRT-TYR subgroup and its binding site motifs are enriched in this group, suggesting that OTX2 is a master transcription regulator for the tyrosinase subgroup [27, 48]. OTX2 demonstrates elevated JSD over the promoter and profound mean hypomethylation consistent with increased expression (Fig. 2c). Interestingly, OTX2 also exhibits a marked reduction in NME compared to control tissue, suggesting a deterministic methylation pattern over this gene, potentially indicating that this is a crucial dependency in this ATRT subtype. Interestingly, *OTX2* is identified by high JSD across all three subtypes, in 3 of 4 ATRT-MYC samples and 8 of 10 ATRT-SHH samples. This suggests that perturbations in the ATRT methylome may converge on common genes that were previously thought to be subtype specific.

**Fig. 3:**
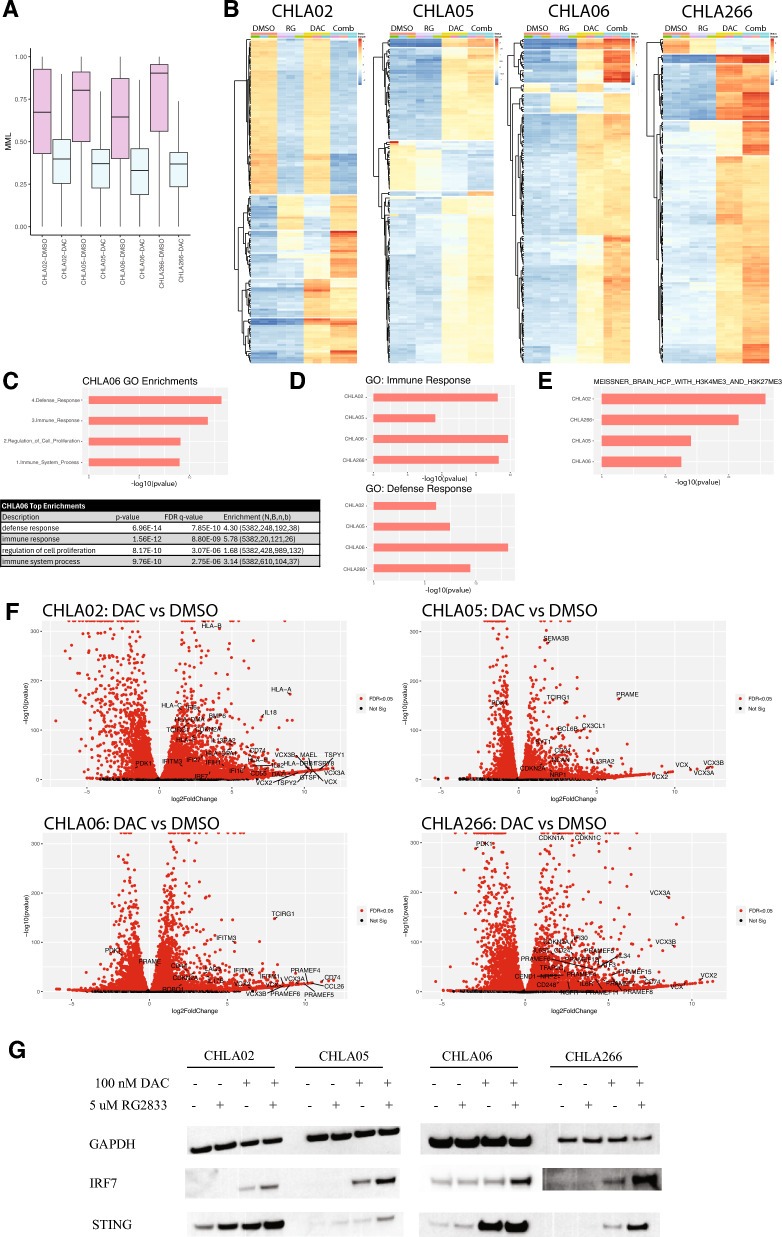
Decitabine reduces DNA methylation levels and synergizes with RG2833 to modulate gene expression. **A** Boxplots depicting genome-wide MML in DMSO (pink) and DAC (blue) for 4 ATRT cell lines. From left-to-right: CHLA02, CHLA05, CHLA06, and CHLA266. Center line represents the median, box is the interquartile range (IQR), and whiskers are 1.5 × IQR. **B** Heatmaps showing the top 150 most differentially expressed genes in 4 ATRT cell lines (from left to right: CHLA02, CHLA05, CHLA06, and CHLA266). Treatment order is as follows from left to right: DMSO, 5 µM RG2833, 100 nM DAC, combination). All heat maps are divided into 5 clusters. Red, upregulated; blue, downregulated. **C** Top 4 Gene Ontology enrichments from GOrilla in CHLA06 for all genes upregulated in combination treatment relative to DMSO. All enrichments were significant (gene ontology, y-axis; -log10(p-value), x-axis) (top graph). *P*-values, FDR q-values and enrichment scores are listed in the bottom table for the top 4 enrichments. N, total number of genes in set; B, total number of genes associated with a gene ontology term; n, number of genes in the top of the upregulated gene set list; b—number of intersecting genes; enrichment = (b/n)/(B/N). **D** Immune response (top) and defense response (bottom) enrichments from Gorilla for all 4 ATRT cell lines, from top to bottom: CHLA02, CHLA05, CHLA06, and CHLA266 (cell line, y-axis; -log10(p-value), x-axis). **E** “MEISSNER_BRAIN_HCP_WITH_H3K4ME3_AND_H3K27ME3” enrichments from GSEA for all 4 ATRT cell lines: CHLA02, CHLA05, CHLA06, and CHLA266 (cell line, y-axis; -log10(p-value), x-axis). **F** Volcano plots of differentially expressed genes for all 4 cell lines (CHLA02, top left; CHLA05 top right; CHLA06, bottom left; CHLA266, bottom right) in DMSO vs. combination treatment comparisons. Universally upregulated genes include VCX/VCX2/VCX3A/VCX3B, and CDKN2A. PDK1 is universally downregulated. Genes are represented as red points if the FDR < 0.05. **G** Western blots of IRF7 (top) and STING (bottom) protein expression in 4 cell lines. From left to right: CHLA02, CHLA05, CHLA06, and CHLA266. GAPDH is used as the loading control. Treatment conditions from left to right are: DMSO, 5µM RG2833, 100 nM DAC, combination

Another gene identified by evaluating DNA methylation stochasticity is *Lin28a*, exhibiting a dramatic increase in JSD, dMML, and dNME directly over the promoter region (Fig. 3d). Importantly, *Lin28a* has been reported to be expressed at high levels in ATRT cell lines and knock-down of this gene leads to reduced growth and induces apoptosis [54]. *Lin28a* is appreciated as a stem cell factor, and thus we hypothesize that elevated entropy may allow distinct populations in the tumor to sample different expression levels of this factor, promoting dedifferentiation or stem cell-like states within the tumor.

Another functionally important gene identified as the target of altered methylation stochasticity across all ATRT subtypes is the prominent tumor suppressor *CDKN2a*. In all three of our representative samples, the *CDKN2a* locus demonstrates elevated JSD and mean hypermethylation (Fig. 2e). This peak of JSD and dMML appears in a low background of JSD and dMML signal, focused specifically on the *CDKN2A* locus. *CDKN2A* is a cell cycle regulator that mediates progression of the cell cycle past the G1 checkpoint and is frequently deleted or silenced via methylation in cancer, including diffuse midline glioma (DMG), where it has been shown in murine models to be downregulated by the K27M mutation [8, 35, 40]. Here, we show that *CDKN2A* is regulated by DNA hypermethylation, likely contributing to gene repression. Analysis of 54 clinically diagnosed primary patient samples published on the PedcBioPortal from the Pediatric Brain Tumor Atlas (PBTA) shows that *CDKN2A* rarely has structural variants in ATRT, with only 2% of samples having a deep deletion [4, 11, 20]. We hypothesize that *CDKN2A* may be repressed in ATRT through DNA hypermethylation.

To identify genes with JSD driven by differential methylation entropy rather than mean changes, we also generated a relative JSD (rJSD) ratio for all genes across these 14 ATRT samples using previously published methods [26] (Supplementary Table 4). The rJSD ratio allows for the identification of genes that are ranked highly by JSD, but have low absolute values of dMML, thus prioritizing genes whose JSD is driven by changes in methylation stochasticity rather than mean methylation levels. For example, in sample ATRT-893, the top gene by rJSD ratio is *SOX11*, which has been identified as a specific marker of the SHH subgroup of ATRT [45]. This gene is ranked #35 by JSD, but #2566 by dMML. Differential methylation over this gene in ATRT-893 is largely driven by changes in entropy rather than MML. As a counterexample, HOXA5 is ranked #1 by JSD and #2 by dMML, indicating that its JSD is primarily driven by mean methylation changes.

We investigated enrichments among genes highly ranked by rJSD across samples. Focusing on the top 200 genes by rJSD, we used GSEA C2 and C5 gene sets to identify enrichments. This revealed enrichments for PRC2, transcription factors, and chromatin regulators consistently across samples, regardless of subtype (Supplementary Table 5) [36, 44]. Additionally, when looking at the overlap of high rJSD genes across samples, key genes related to embryogenesis and neuronal differentiation appear near the top, including *TBXT*, *SALL1*, *POU3F3*, and *PRDM14,* among others (Supplementary Table 4). Interestingly, these genes have not emerged as key targets or drivers in ATRT and have not been reported in other literature describing altered mean methylation levels in ATRT. Further work will be needed to assess for functional impact of high rJSD genes in ATRT, motivated by prior literature showing that elevated methylation entropy is related to variability of gene expression.

### Pharmacologic targeting of the ATRT methylome with hypomethylating agents modulates methylation patterns over key regions

Given the aberrant methylation landscape in ATRT, with both subgroup-specific alterations and common features such as focal hypermethylation and increased methylation entropy over key regulatory elements, we hypothesized that this methylation pattern was selected to support a favored set of gene expression states that promote tumorigenesis. We aimed to modulate this methylation pattern pharmacologically by first employing the DNA methyltransferase inhibitor (DNMTi), decitabine (DAC). Decitabine is a cytidine analog in which the carbon at position 5 of the cytosine ring is replaced by nitrogen. It is incorporated into the growing DNA strand during S phase and traps DNA methyltransferase enzymes (DNMTs), thus leading to genome-wide hypomethylation after subsequent rounds of cell division [25]. At high doses, it acts as an antimetabolite; however, at low doses it causes limited DNA damage and has been shown to induce differentiation [28]. Clinically meaningful “low dose” decitabine ranges from 20–300 nM [49].

We hypothesized that ATRT would be exquisitely sensitive to agents that perturb the preferred balance of methylation, specifically as hypermethylated regulatory regions and tumor suppressor genes become hypomethylated. This disruption in methylation is hypothesized to have downstream consequences for the expression of genes under the regulation of bivalent promoters and differentiation programs.

**Fig. 4:**
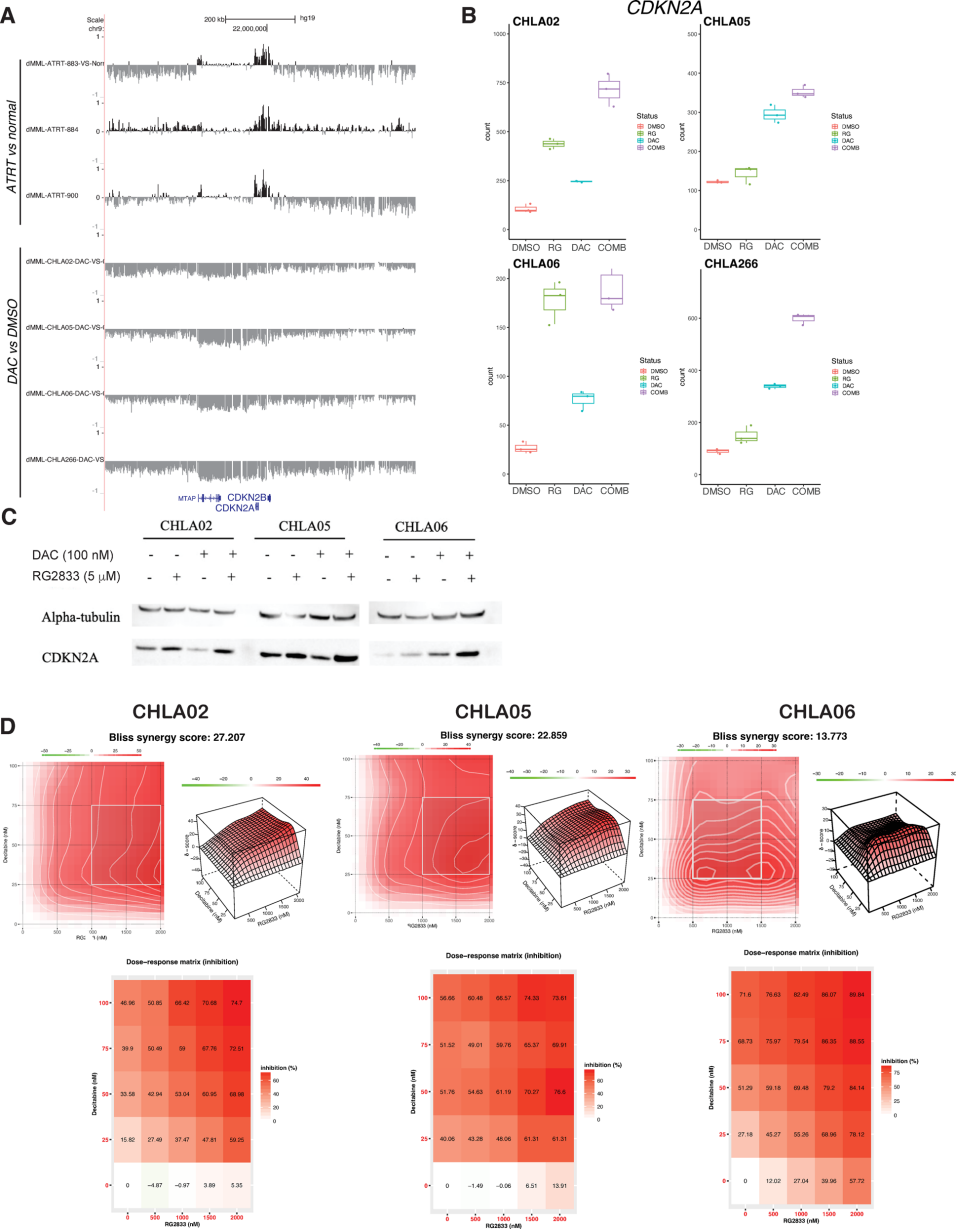
DAC and RG2833 upregulate the expression of CDKN2A and lead to a reduction in cell viability. **A** GBiB tracks of dMML of ATRT-883, ATRT-884, and ATRT-900 for ATRT vs. Normal comparisons over the *CDKN2A* locus, replicated from Fig. 2e for comparison (top). GBiB tracks of dMML for CHLA02, CHLA05, CHLA06, and CHLA266 vs. DMSO treatment over the *CDKN2A* locus (bottom). **B** Box plots demonstrating mRNA count (n = 3) for CHLA02, CHLA05, CHLA06, and CHLA266. Treatment conditions are DMSO (red), 5 µM RG2833 (green), 100 nM DAC (blue), and combination (purple). Center line, median; box; IQR, whiskers: 1.5 × IQR. **C** Western blots of CDKN2A protein expression in CHLA02, CHLA05, and CHLA06. GAPDH is used as the loading control. Treatment conditions from left to right are DMSO, 5 µM RG2833, 100 nM DAC, combination. **D** Bliss synergy results from treatment of CHLA02, CHLA05, and CHLA06 with escalating concentrations of DAC (0 nM, 25 nM, 50 nM, 75 nM, 100 nM) and/or RG2833 (0 nM, 500 nM, 1 µM, 1.5 µM, and 2 µM). Shown are dose–response density graphs, 3D response plots, and a dose–response matrix based on inhibition (x-axis = RG2833, y-axis = decitabine)

We performed WGBS on 4 patient-derived cell lines of ATRT, CHLA02, CHLA05, CHLA06, and CHLA266, following a 5-day treatment with 100 nM DAC and applied the informME analysis pipeline to characterize methylation patterning across the genome. CHLA02 and CHLA05 are of the ATRT-SHH subtype, and CHLA06 and CHLA266 are of the ATRT-MYC subtype. Decitabine treatment led to a dramatic reduction in MML genome-wide in all cell lines (Fig. 4a). Globally, this was accompanied by an increase in NME, presumably due to random demethylation of a genome that was largely methylated at baseline, resulting in more methylation patterns (Supplementary Figs. 2a-b). ChromHMM classes exhibiting focal hypermethylation in ATRT are effectively hypomethylated following decitabine treatment (Supplementary Fig. 2c). This responsiveness of the ATRT methylation landscape to brief decitabine treatment highlights the dynamic nature and active remodeling of the ATRT methylome. We next sought to assess the functional impacts of decitabine-induced demethylation, in combination with other agents that modify the epigenome, on gene expression.

### Pharmacologic modulation of DNA methylation in ATRT cell lines alters gene expression

Our results highlight the dynamic and disordered methylome of ATRT and suggest that methylation alterations can be readily reversed with decitabine. Prior work has also shown a unique chromatin landscape in ATRT. At promoter regions, ATRT has a reduction in the activating H3K27ac mark, which is not met with an increase in the repressive H3K27me3 mark but rather is met with a local increase in DNA methylation [15]. We therefore hypothesized that ATRT would be sensitive to combination treatment with decitabine and HDAC inhibition (HDACi). We used RG2833, a brain-penetrant HDACi that is selective for HDAC classes 1/3. Additionally, hypomethylation and hyperacetylation (alone and in combination) have previously been shown to reactivate endogenous retrovirus elements and enhance innate immune signaling pathways in ovarian cancer and diffuse midline glioma, suggesting that the combination may enhance the immunogenicity of ATRT [5, 31].

Continuing with low-dose DAC treatment in ATRT cell lines, we next assessed the addition of RG2833 following 5 days of decitabine pre-treatment, evaluating gene expression by RNA-seq at the 72-h time point. Principal Component Analysis (PCA) revealed tight clustering and separation of the DMSO, DAC, RG2833, and combination treatment groups across all 4 cell lines (Supplementary Fig. 3). Heatmap analysis of the top differentially expressed genes in each cell line after treatment with DMSO, RG2833, DAC, or the combination of DAC and RG2833 reveals clusters that separate according to treatment (Fig. 3b, Supplemental Fig. 4 [with gene names]). While a notable portion of the upregulation was driven by DAC monotherapy, there were unique clusters in all cell lines where the combination treatment further enhanced gene expression alterations.

In all, combination treatment compared to control led to widespread changes in gene expression. GOrilla gene ontology analysis on all upregulated genes in CHLA06 combination treatment revealed that the 4 most enriched gene ontology sets were “defense response,” “immune response,” “regulation of cell proliferation,” and “immune system process” [13, 14]. All enriched sets had a *p*-value of less than 9.5e−10 (Fig. 3c). Exploration of the other 3 cell lines also revealed enrichment for the “immune response” and “defense response” gene sets, with p-values less than 7.8e−7 for these sets (Fig. 3d). The top 4 GOrilla enrichment sets for the remaining cell lines are shown in supplementary Fig. 5a. Full enrichment sets are listed in Supplementary Table 5.

Interestingly, we observed that all cell lines had highly significant enrichments for “cell adhesion,” “cell migration,” and “extracellular matrix organization” (Supplementary Table 5). Graf et al. observed similar enrichment sets for upregulated genes after treatment of BT16, an ATRT-MYC line, with long-term (14 day), low micromolar dose (0.5 uM) decitabine. Using data from Wang et al., they also show that similar enrichments are seen in BT16 after long-term treatment with DAC as with SMARCB1 reactivation. They report that “cell adhesion,” “cell migration,” and “extracellular matrix organization” are the top 3 most significantly enriched pathways after SMARCB1 reactivation [22, 53]. Additionally, Wang et al. published GSEA enrichments for upregulated genes after SMARCB1 re-expression [53]. One set that was present in their data set after SMARCB1 reactivation and in 2 of our cell lines, CHLA02 and CHLA05, after combination treatment was “WANG_SMARCE1_TARGETS_UP,” which represents genes that are upregulated in breast cancer with reintroduction of SMARCE1, another component of SWI/SNF (Supplementary Fig. 5b). Many of the genes in this set are reported to oppose cell cycle progression and induce apoptosis [52]. We also find that upregulated genes after combination treatment are enriched for genes under the regulation of bivalent promoters and/or PRC2, as shown by enrichments for “NUYTTEN_EZH2_TARGETS_UP” and “MEISSNER_BRAIN_HCP_WITH_H3K4ME3_AND_H3K27ME3” (Supplementary Fig. 5c, Fig. 3e) based on C2 GSEA results (Supplementary Table 5) [34, 39]. We have already shown here that genes with the highest methylation stochasticity are genes under the regulation of PRC2 and bivalent promoters, and we now show that the expression of these genes is profoundly affected by treatment with decitabine.

Key genes enriched in the “Defense Response” and “Immune Response” sets are highlighted in the volcano plots in Fig. 3f. All 4 cell lines demonstrated strong upregulation for the human leukocyte antigen (HLA), interleukin, interferon, and interferon-induced transmembrane genes. CHLA02, CHLA05, and CHLA266 all had upregulation of *CD74.* It has been shown that CD74 expression is predictive of dual PDL-1 and CTLA-4 immune checkpoint blockade therapy response [51]. Additionally, *IL13RA2* is upregulated in both CHLA02 and CHLA05. IL13RA2 is a target of cell-based immunotherapies, and a phase I clinical trial of IL13RA2 directed CAR-T cells for recurrent high-grade glioblastoma was recently completed (NCT02208362). Analysis of other gene set enrichment lists in CHLA05 also demonstrated an enrichment in genes related to neuronal differentiation, including *SEMA3B* and *SYT1*, and tumor suppressor genes, such as *BCL6B*, which are also displayed in Fig. 3f. Additionally, *LAG3* was upregulated in both CHLA06 and CHLA266, and *CD274* was upregulated in CHLA266. Both LAG3 and CD274 are cell surface molecules that are the targets of existing immunotherapies (NCT02658981).

Genes that are universally increased across all 4 cell lines include *CDKN2A* and the VCX family of genes *(VCX*, *VCX2*, *VCX3A*, *VCX3B*) (Fig. 3f). The VCX family genes are cancer testis antigens (CTAs), which are proteins that are not normally expressed in adult tissues, except in male germ cells, but can be expressed on cancer cells and are highly immunogenic [19]. Additionally, *CD40*, a target of immunotherapy in ATRT clinical trials, also experienced profound upregulation with combination treatment, though expression levels in our samples were low at baseline (NCT03389802). Alternatively, *PDK1*, which is an inhibitor of Pyruvate Phosphate Dehydrogenase, was universally downregulated across all 4 cell lines. PDK1 is a downstream target of the PI3K/AKT pathway, a pathway which has been shown to be activated in cells that lack SMARCB1 activity and is implicated in ATRT tumorigenesis [9].

We also show by western blot that DAC and RG2833 increase the expression of Stimulator of Interferon Genes (STING) and Interferon Regulatory Factor 7 (IRF7) (Fig. 3g). STING is activated by cGAS during the detection of cytosolic nucleic acids, and its activation leads to the downstream upregulation of interferon stimulatory genes and type I interferons [24]. Additionally, IRF7 is a transcription factor that responds to foreign nucleic acids in the cell and activates type I interferons and other innate immune signaling pathways [38]. We also confirm the upregulation of IFITM3 at the protein level in CHLA02 and CHLA06, the two cell lines that had upregulation at the RNA-seq level (Supplementary Fig. 5d).

Taken together, we corroborate the previous finding that decitabine can upregulate gene pathways that are seen with SMARCB1 reactivation. We also demonstrate that the combination of DNMTI, decitabine, and the HDACi, RG2833, induces profound upregulation of genes that are under the regulation of PRC2 and bivalent promoters. We also show upregulation of CTAs, genes related to interferon signaling, interferon induced transmembrane proteins, and HLA genes across individual samples and subgroups (ATRT-MYC and ATRT-SHH). It also leads to increased expression of relevant immunotherapy targets such as *PD-L1, LAG-3*, and *CD40*. Further work in immunocompetent in vivo models will be necessary to assess whether this upregulation in neoantigens, HLA genes, and innate immune signaling pathways can be leveraged to improve antitumor immune responses.

### Decitabine and RG2833 treatment leads to re-expression of *CDKN2A* and synergistically reduce cell viability

We next evaluated the effects of DAC and RG2833 on the expression of *CDKN2A.* It has been shown in other cancers that demethylation at the *CDKN2A* promoter can reverse the downregulation commonly seen in cancers lacking *CDKN2A* deletion [8, 35]. Figure 4a contrasts the dMML tracks of primary ATRT samples versus normal (showing CDKN2a hypermethylation), with dMML of ATRT cell lines treated with decitabine vs. DMSO vehicle (showing profound hypomethylation of the same region). Accordingly, RNA-Seq data confirm CDKN2a re-expression by DAC/RG2833 treatment in all cell lines (Fig. 4b). In all cell lines, *CDKN2A* expression is highest in the combination treatment. We confirmed treatment-induced CDKN2A re-expression at the protein level (Fig. 4c).

Since DAC/RG2833 treatment induced re-expression of CDKN2A in addition to restoration of pathways similarly re-expressed with *SMARCB1* knock-in, we investigated the effect of this combination treatment on cell viability. We evaluated sensitivity of three ATRT cell lines, CHLA02, CHLA05, and CHLA06, to varying concentrations of decitabine or vehicle, followed by RG2833 or vehicle, assessing viability by flow cytometry. Synergy between the two agents was evaluated using the Bliss synergy score, with a score above 10 considered synergistic [23]. The scores for CHLA02, CHLA05, and CHLA06 were 27.207, 22.859, and 13.773, respectively, suggesting that decitabine and RG2833 are highly synergistic in combination (Fig. 4d). Given large-scale gene expression changes between control and DAC/RG2833 combination treatments, it is likely that the synergistic reduction in cell viability results from effects on multiple targets rather than re-expression of a single gene or pathway, but further studies to investigate the mechanism of this synergy will be necessary.

## Discussion

Despite the low mutational burden of ATRT aside from SMARCB1 loss, there is extensive intertumoral heterogeneity in gene expression profiles and chromatin landscapes, and 3 distinct disease subgroups [27]. We sought to apply a novel analysis pipeline to WGBS of primary ATRT patient samples to evaluate stochastic DNA methylation variation in individual ATRT methylomes and across ATRT subgroups. Since DNA methylation variability can underlie phenotypic plasticity and intratumoral heterogeneity, this analysis fills a gap in our understanding of ATRT biology. We find broad changes in mean methylation and methylation entropy between subgroups, but also common focal alterations, such as elevated mean methylation and entropy over bivalent promoters.

Bivalent promoters can be dually occupied by the activating H3K4me3 mark and the repressive H3K27me3 mark. SMARCB1 acts antagonistically towards PRC2, the complex that deposits the H3K27me3 mark, and so loss of SMARCB1 can lead to unchecked activity of PRC2. Further work is needed to clarify the mechanisms by which aberrant PRC2 activity in the absence of SMARCB1 contributes to locally altered methylation entropy at these regions [55]. 

Genes exhibiting altered methylation stochasticity in ATRT were highly enriched for targets of PRC2 and bivalent promoters. Highly stochastic genes were also enriched for gene sets related to organ morphogenesis, differentiation, and the nervous system. Given that ATRT is an undifferentiated embryonal tumor, it is plausible that pathways related to differentiation and morphogenesis are under the regulation of stochastic methylation patterns. Additionally, we show that methylation stochasticity is highest over cell cycle regulators and known drivers of ATRT, including the *HOXD* cluster, *OTX2*, and *LIN28a* [27, 54]. This suggests that known drivers of oncogenesis are under plastic methylation regulation, affording the opportunity for distinct transcriptional regulation.

Additionally, our rJSD analysis identifies genes that have high methylation stochasticity, but do not differ significantly in their mean levels of methylation. Such genes would be missed by conventional analysis methods focused solely on mean methylation [26]. Prior work has shown that JSD is not always concordant with absolute values of dMML, and use of JSD rather than mean methylation leads to improved identification of differentially methylated genes [30]. Additionally, integration of WGBS and single-cell data sets have previously shown that high methylation entropy, rather than mean methylation, is correlated with cell-to-cell gene expression variability in cancer and developmental contexts [17, 30]. It is interesting that these analyses identify enrichments for genes in differentiation pathways, given that cancer encounters a block to normal lineage determination. This suggests a model in which cells are variably sampling expression states of key genes related to stemness, and this variability is modulated by DNA methylation patterning. Further single-cell work will be needed to determine if this relationship between methylation entropy and gene expression variability also holds true for ATRT. Though high methylation entropy has been reported as a mechanism of transcriptional plasticity in cancer and development, further studies are also necessary to elucidate the link between altered epigenetic drivers such as SMARCB1 loss and downstream changes in methylation entropy.

Following our molecular characterization of the ATRT methylome, we then assessed the responsiveness of 4 ATRT cell lines, CHLA02, CHLA05, CHLA06, and CHLA266, to pharmacologic modulation of the epigenome. We were interested in investigating the efficacy of decitabine given that our data from the primary patient samples suggested that there are genome-wide perturbations in methylation patterns in ATRT that also map focally onto bivalent promoters, genes under the regulation of PRC2, and genes that are known drivers of ATRT. Many of these key focal perturbations involve hypermethylation, which we hypothesized would be sensitive to hypomethylation with decitabine. We demonstrate with WGBS that DAC can hypomethylate ATRT cell lines globally and focally at key regulatory regions, such as bivalent promoters and certain enhancer classes. We did not investigate pharmacological manipulation of the epigenome in an ATRT cell line of the tyrosinase subgroup. Our WGBS analysis of our primary patient samples shows that ATRT-TYR is hypermethylated globally, and this pattern persists at local regulatory regions, such as bivalent promoters, and PRC2-dependent genes. Given this, we hypothesize that ATRT-TYR cell lines will also be sensitive to pharmacological manipulation of their epigenome, similar to ATRT-MYC and ATRT-SHH.

We next assessed how the addition of the HDACi, RG2833, would affect gene expression in combination with decitabine. This combination was motivated by previous reports that ATRT has aberrant H3K27ac downstream of SMARCB1 inactivation, that is met with elevated DNA methylation over these sites, and that hypomethylation and hyperacetylation can elicit innate immune signaling in other tumor types [5, 15, 31]. Here, we report that DAC and RG2833 potently increase immune signaling in ATRT cell lines, with induction of STING/interferon pathways, CTAs, and HLA related genes. Given very low MHC class I expression in ATRT, the finding that DNMTi and HDACi induce upregulation of HLA class I genes across subtypes may be highly relevant to future studies. Interestingly, we also noted profound upregulation of *VCX3A*, which has been reported to regulate the expression of HLA molecules in diffuse midline glioma [10].

Additionally, our combination therapy also led to the reactivation of numerous cell surface molecules, such as *CD40* and *PD-L1,* at the mRNA level that are targets of existing clinical trials in ATRT. Previous clinical trials using PD-L1 blockade monotherapy in ATRT were terminated due to low efficacy, and new clinical trials are aimed at combining PD-L1 blockade with TIGIT blockade [43] (NCT05286801). Our findings suggest that a strategy employing epigenetic pre-treatment prior to immunotherapy in ATRT has promise and warrants further preclinical investigation using immunocompetent in vivo models. 

We also report here that dual treatment with the DNMTi, decitabine, and the HDACi, RG2833, leads to the re-expression of the tumor suppressor, CDKN2A, at the mRNA and protein level. Interestingly, synergistic reactivation of *p16* has been demonstrated using decitabine and an HDACi, such as trischostatin A (TSA), in other cancers, including head and neck squamous cell carcinoma (HNSCC) [3, 6]. In HNSCC, for example, there was moderate increase in H3K27ac at the promoter with decitabine alone and only a slight increase in histone acetylation ahead of the *CDKN2A* promoter with TSA alone, but a striking increase when TSA and decitabine were used in combination. This supports the hypothesis that reduced CpG methylation may be necessary for histone acetylation at the *CDKN2A* promoter leading to re-expression of silenced *CDKN2A*. The finding that demethylation is necessary for effective hyperacetylation by HDACi was also reproduced more recently in lung cancer models, in the context of inducing de novo transcription from cryptic transcription start sites [2]. It is possible that an analogous mechanism applies to ATRT, whereby drug-induced DNA hypomethylation promotes acetylation by HDAC inhibitors at promoters of genes silenced in ATRT cells. Further work assessing distribution of the H3K27ac mark would be necessary to clarify this mechanism in ATRT.

Additionally, we corroborated findings from Graf et al. based on data generated by Wang et al. that decitabine, here in combination with RG2833, leads to the upregulation of gene sets that are also upregulated upon wild-type SMARCB1 introduction in rhabdoid tumors [22, 53]. We also show reactivation of genes under the regulation of bivalent promoters and PRC2. We hypothesize that re-expression of tumor suppressor genes and genes that are normally silenced by SMARCB1 inactivation act contribute to reduced cell viability.

Taken together, we provide promising evidence that the highly disordered DNA methylome of ATRT is responsive to epigenetic therapies, leading to dramatic alterations in gene expression including the reactivation of innate immune system pathways, tumor suppressors, and SMARCB1 dependent pathways. In vivo experiments will be required to validate the efficacy of this combination for ATRT. Encouragingly, flank tumors of malignant rhabdoid tumor have responded favorably to decitabine treatment, suggesting the potential of hypomethylating agents for rhabdoid tumor [22]. Importantly, however, these experiments were conducted using immunocompromised mouse models, and so we hypothesize that the reduction in tumor volume will be augmented in an animal model with an intact immune system, given the prominent upregulation of innate immune signaling reported here. Our findings support a compelling hypothesis that immune modulation by epigenetic therapy can enhance ATRT antitumor immunity, providing strong rationale for future studies in immunocompetent models.

## Supplementary Information

Supplementary Material 1. Figure 1. WGBS analysis for clinically diagnosed ATRT primary patient samples over core genomic regions. A) Box-plots depicting differential MML (dMML) (left), differential NME (dNME) (middle), and Jensen-Shannon distance (JSD) (right) observed for normal-1/ATRT-883 (top), normal-1/ATRT-884 (middle), and normal-1/ATRT-900 (bottom) comparisons genome-wide, and within CpG islands, CpG shores, CpG shelves, CpG open seas, gene bodies, exons, introns, and intergenic regions. Center line is the media, box is the IQR and whiskers are 1.5 x IQR. B) Boxplots of dMML (left), dNME (center), JSD (right) for 25 ChromHmm genomic annotations in ATRT-884 (top) and ATRT-900 (bottom). Center line is the media, box is the IQR and whiskers are 1.5 x IQR. C) Smoothed MML (top) and NME (bottom) over genomic regions +/- 2kb from bivalent promoters for control (blue) and ATRT samples (pink) for ATRT-883 (top), ATRT-884 (middle), and ATRT-900 (bottom).

Supplementary Material 2. Figure 2. WGBS analysis for decitabine treated ATRT patient-derived cell lines over core genomic regions. A) Boxplots of genome-wide NME in DMSO (pink) and DAC (blue) for 4 ATRT cell lines: CHLA02, CHLA05, CHLA06, and CHLA266. Center line represents the median, box is the interquartile range (IQR), and whiskers are 1.5 x IQR. B) Line plots showing MML (top) and NME (bottom) for DMSO (blue) and DAC (pink) +/- 2 kb from the TSSs genome-wide in CHLA02. C) Boxplots showing dMML (left), dNME (middle), and JSD (right) observed for DMSO/DAC comparisons across 12 ChromHmm annotation sites in CHLA02, CHLA05, CHLA06, and CHLA266 (bottom).

Supplementary Material 3. Figure 3. Clustering of ATRT cell lines after treatment with hypomethylating and hyperacetylating agents. PCA plots of CHLA02 (top left), CHLA05 (top right), CHLA06 (bottom left) and CHLA266 (bottom right) following treatment with DMSO (blue circles), RG2833 (purple cross), decitabine (green square), and combination treatment (orange triangle)

Supplementary Material 4. Figure 4. Heatmaps of ATRT cell lines after treatment with DAC and RG2833. Heatmaps from figure 3b reshown with the gene names listed. Depicts the top 150 most differentially expressed genes in 4 ATRT cell lines: CHLA02, CHLA05, CHLA06, and CHLA266). Treatment order is as follows from left to right: DMSO, 5µM RG2833, 100 nM DAC, combination). All heat maps are divided into 5 clusters. Red, upregulated; blue, downregulated.

Supplementary Material 5. Figure 5. Gene Enrichments in ATRT cell lines after treatment with decitabine and RG2833. A) Top 4 Gene Ontology enrichments from GOrilla in CHLA02, CHLA05, and CHLA266 for all genes upregulated in combination treatment relative to DMSO. All enrichments were significant (gene ontology = y-axis; -log10(p-value) = x-axis). B) “WANG_SMARCE1_TARGETS_UP” enrichments from GSEA for 2 ATRT cell lines: CHLA02 and CHLA05 (cell line, y-axis; -log10(p-value), x-axis). C) “NUYTTEN_EZH2_TARGETS_UP” enrichments from GSEA for all 4 ATRT cell lines: CHLA02, CHLA05, CHLA06, and CHLA266 (cell line, y-axis; -log10(p-value), x-axis). D) Western blots of IFITM3 protein expression in CHLA02 (left) and CHLA06 (right), from the same experiment as presented in figure 4c. GAPDH is repeated here as the loading control. Treatment conditions from left to right are: DMSO, 5 µM RG2833, 100 nM DAC, combination.

Supplementary Material 6.

Supplementary Material 7.

Supplementary Material 8.

Supplementary Material 9.

Supplementary Material 10.

Supplementary Material 11.

## Data Availability

The datasets generated in the current study are available in the Gene Expression Omnibus and Sequence Read Archive repositories, with accession numbers GSE301410 (WGBS data), GSE301405 (RNA-seq data), GSE301412 (WGBS data).
