# Supplementary figures and images for: Disordered DNA methylation leads to targetable transcriptional plasticity in ATRT
Source: Acta Neuropathol Commun. 2025 Dec 17;14:22. doi: 10.1186/s40478-025-02173-y (PMC12821819; doi:10.1186/s40478-025-02173-y)

A

ATRT-883

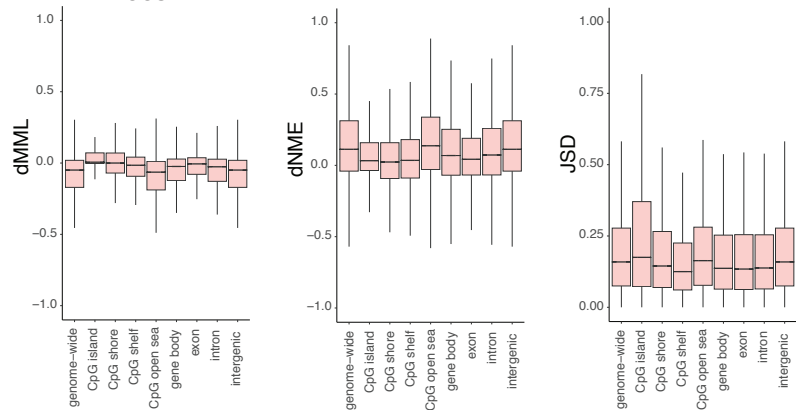

ATRT-884

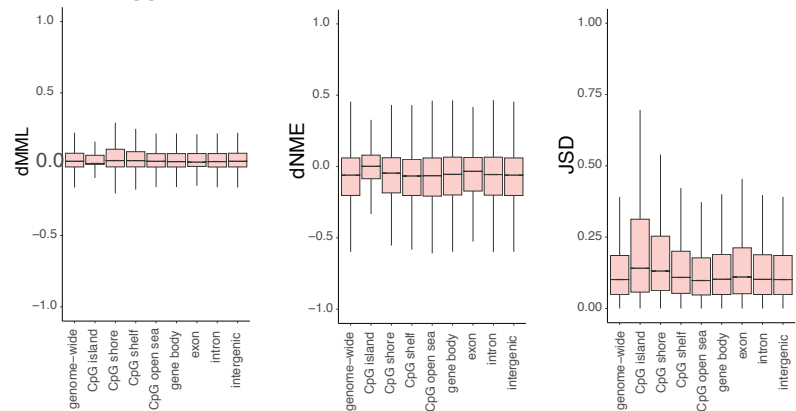

ATRT-900

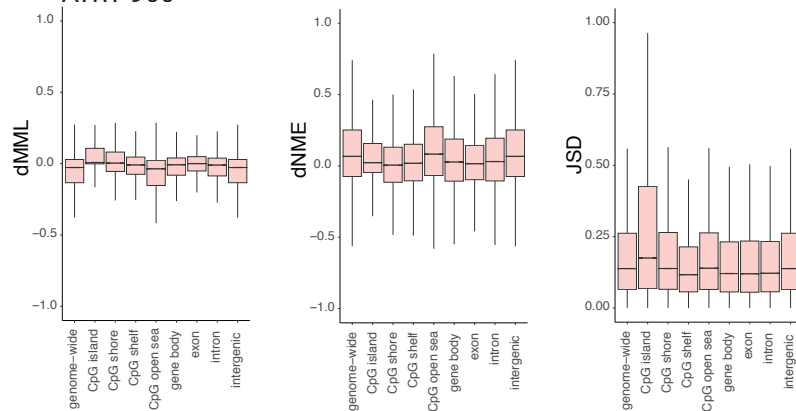

B

ATRT-884

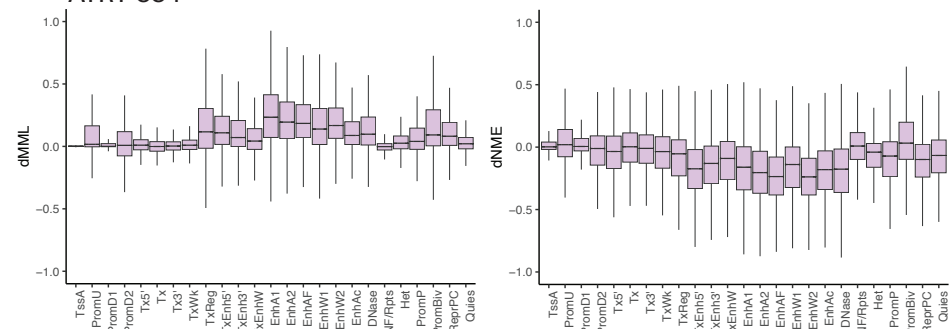

ATRT-900

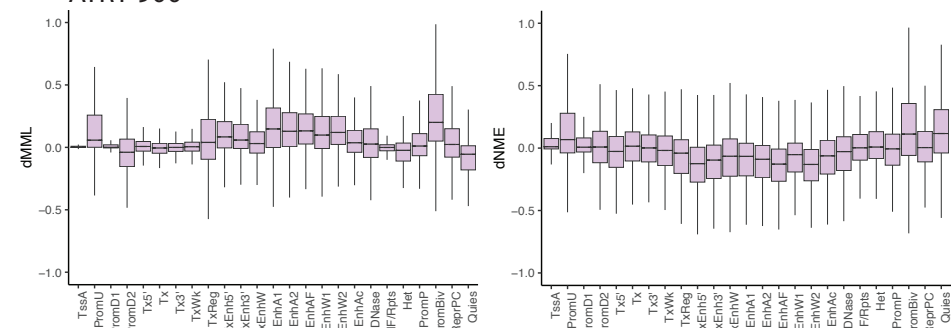

C

ATRT-883

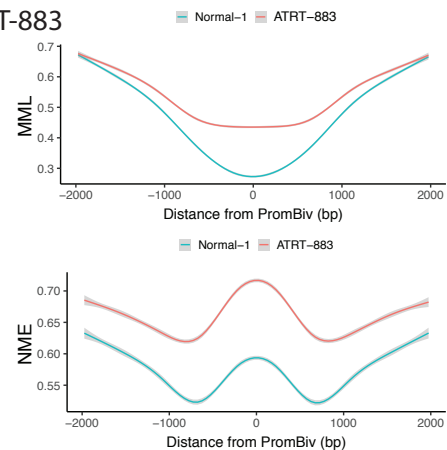

ATRT-884

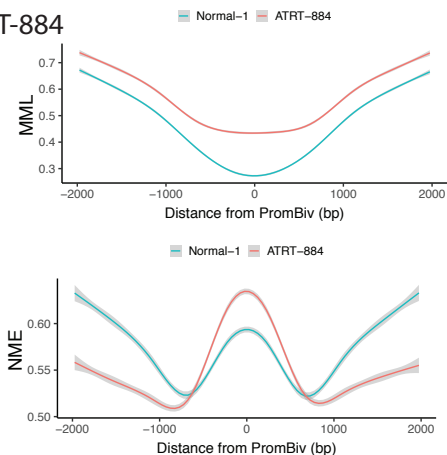

ATRT-900

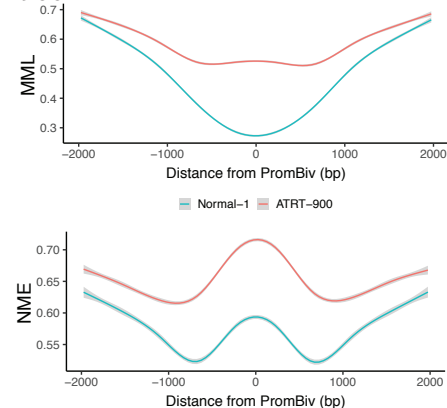

Supplement: Supplementary file 1 — Supplementary Material 1. Figure 1. WGBS analysis for clinically diagnosed ATRT primary patient samples over core genomic regions. A) Box-plots depicting differential MML (dMML) (left), differential NME (dNME) (middle), and Jensen-Shannon distance (JSD) (right) observed for normal-1/ATRT-883 (top), normal-1/ATRT-884 (middle), and normal-1/ATRT-900 (bottom) comparisons genome-wide, and within CpG islands, CpG shores, CpG shelves, CpG open seas, gene bodies, exons, introns, and intergenic regions. Center line is the media, box is the IQR and whiskers are 1.5 x IQR. B) Boxplots of dMML (left), dNME (center), JSD (right) for 25 ChromHmm genomic annotations in ATRT-884 (top) and ATRT-900 (bottom). Center line is the media, box is the IQR and whiskers are 1.5 x IQR. C) Smoothed MML (top) and NME (bottom) over genomic regions +/- 2kb from bivalent promoters for control (blue) and ATRT samples (pink) for ATRT-883 (top), ATRT-884 (middle), and ATRT-900 (bottom). [file 40478_2025_2173_MOESM1_ESM.pdf]

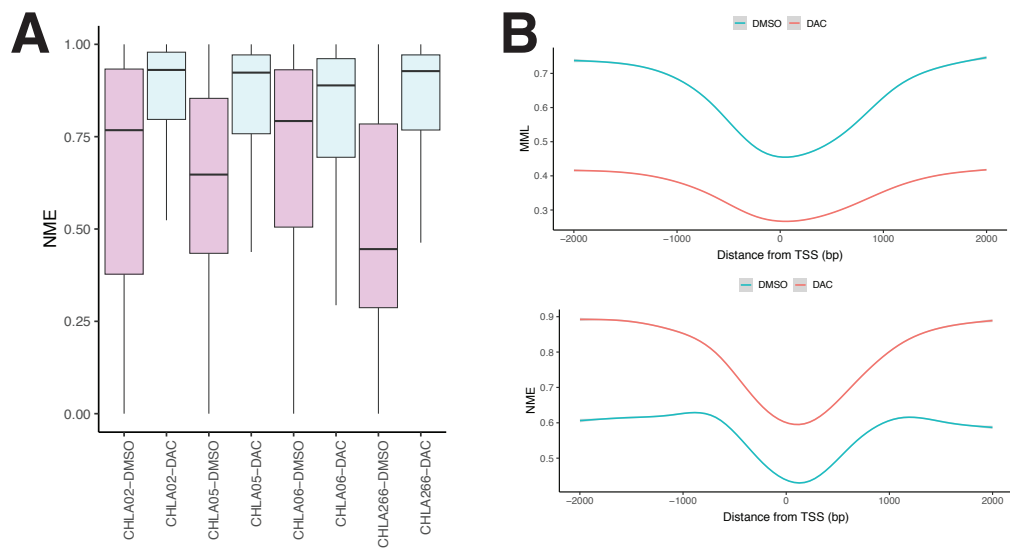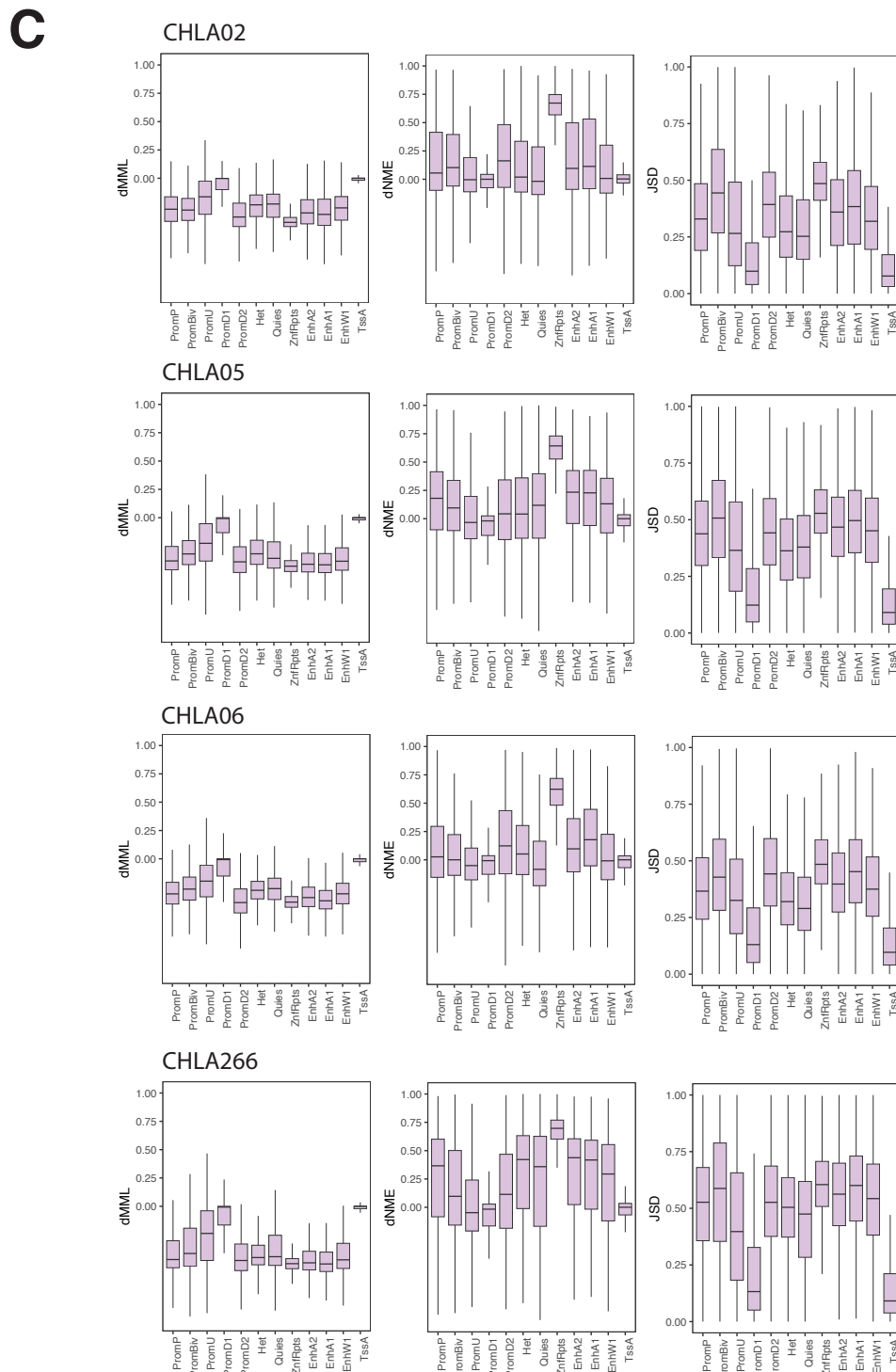

Supplement: Supplementary file 2 — Supplementary Material 2. Figure 2. WGBS analysis for decitabine treated ATRT patient-derived cell lines over core genomic regions. A) Boxplots of genome-wide NME in DMSO (pink) and DAC (blue) for 4 ATRT cell lines: CHLA02, CHLA05, CHLA06, and CHLA266. Center line represents the median, box is the interquartile range (IQR), and whiskers are 1.5 x IQR. B) Line plots showing MML (top) and NME (bottom) for DMSO (blue) and DAC (pink) +/- 2 kb from the TSSs genome-wide in CHLA02. C) Boxplots showing dMML (left), dNME (middle), and JSD (right) observed for DMSO/DAC comparisons across 12 ChromHmm annotation sites in CHLA02, CHLA05, CHLA06, and CHLA266 (bottom). [file 40478_2025_2173_MOESM2_ESM.pdf]

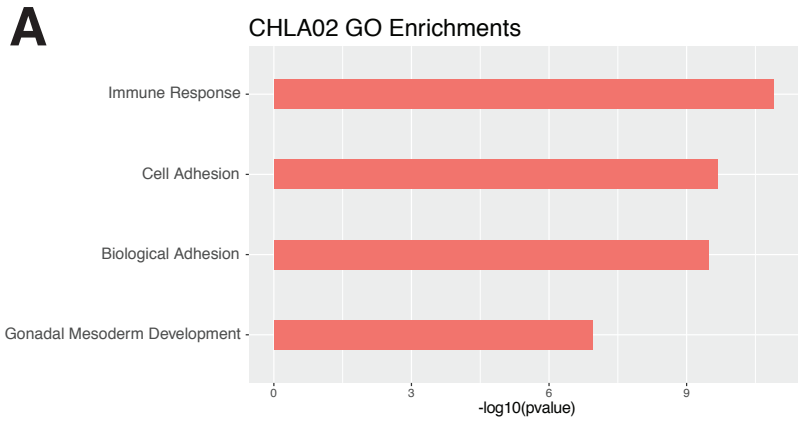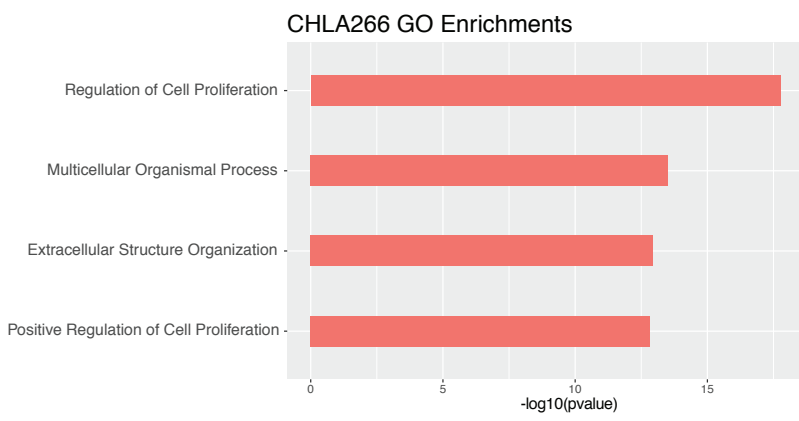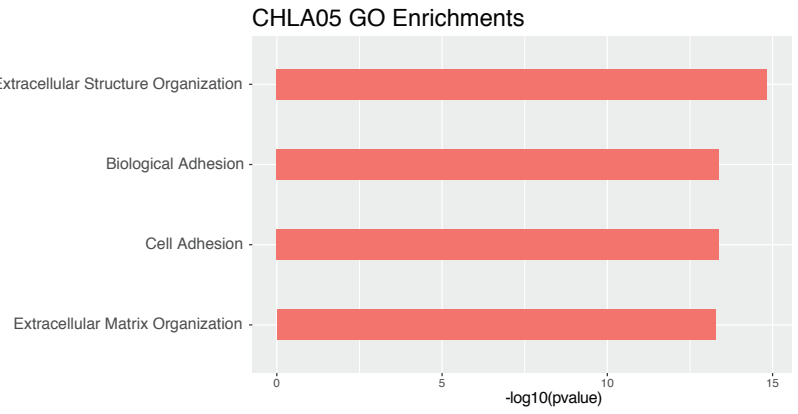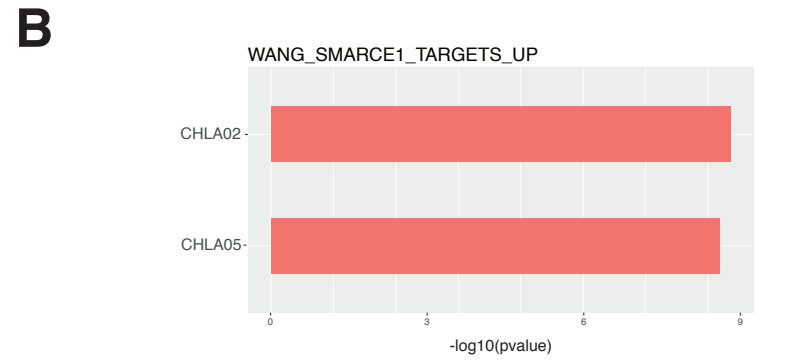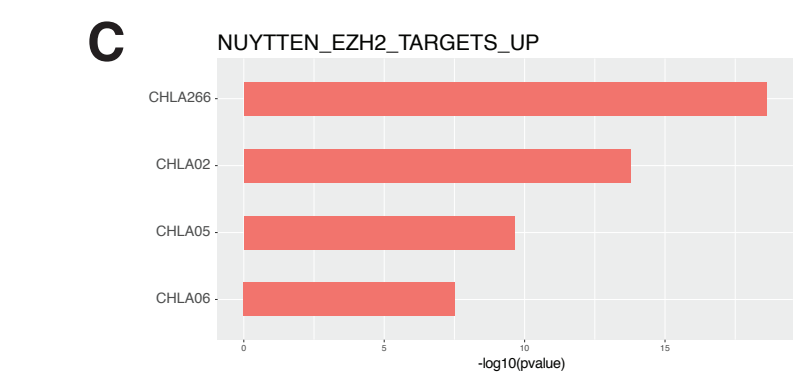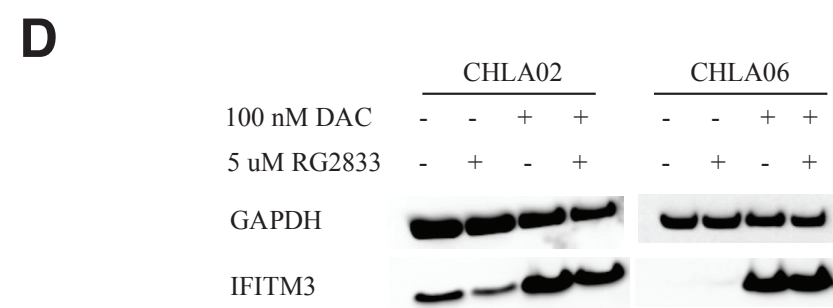

Supplement: Supplementary file 5 — Supplementary Material 5. Figure 5. Gene Enrichments in ATRT cell lines after treatment with decitabine and RG2833. A) Top 4 Gene Ontology enrichments from GOrilla in CHLA02, CHLA05, and CHLA266 for all genes upregulated in combination treatment relative to DMSO. All enrichments were significant (gene ontology = y-axis; -log10(p-value) = x-axis). B) “WANG_SMARCE1_TARGETS_UP” enrichments from GSEA for 2 ATRT cell lines: CHLA02 and CHLA05 (cell line, y-axis; -log10(p-value), x-axis). C) “NUYTTEN_EZH2_TARGETS_UP” enrichments from GSEA for all 4 ATRT cell lines: CHLA02, CHLA05, CHLA06, and CHLA266 (cell line, y-axis; -log10(p-value), x-axis). D) Western blots of IFITM3 protein expression in CHLA02 (left) and CHLA06 (right), from the same experiment as presented in figure 4c. GAPDH is repeated here as the loading control. Treatment conditions from left to right are: DMSO, 5 µM RG2833, 100 nM DAC, combination. [file 40478_2025_2173_MOESM5_ESM.pdf]
