# Supplementary material for: Disordered DNA methylation leads to targetable transcriptional plasticity in ATRT
Source: Acta Neuropathol Commun. 2025 Dec 17;14:22. doi: 10.1186/s40478-025-02173-y (PMC12821819; doi:10.1186/s40478-025-02173-y)
Supplement: Supplementary file 3 — Supplementary Material 3. Figure 3. Clustering of ATRT cell lines after treatment with hypomethylating and hyperacetylating agents. PCA plots of CHLA02 (top left), CHLA05 (top right), CHLA06 (bottom left) and CHLA266 (bottom right) following treatment with DMSO (blue circles), RG2833 (purple cross), decitabine (green square), and combination treatment (orange triangle) [file 40478_2025_2173_MOESM3_ESM.pdf]

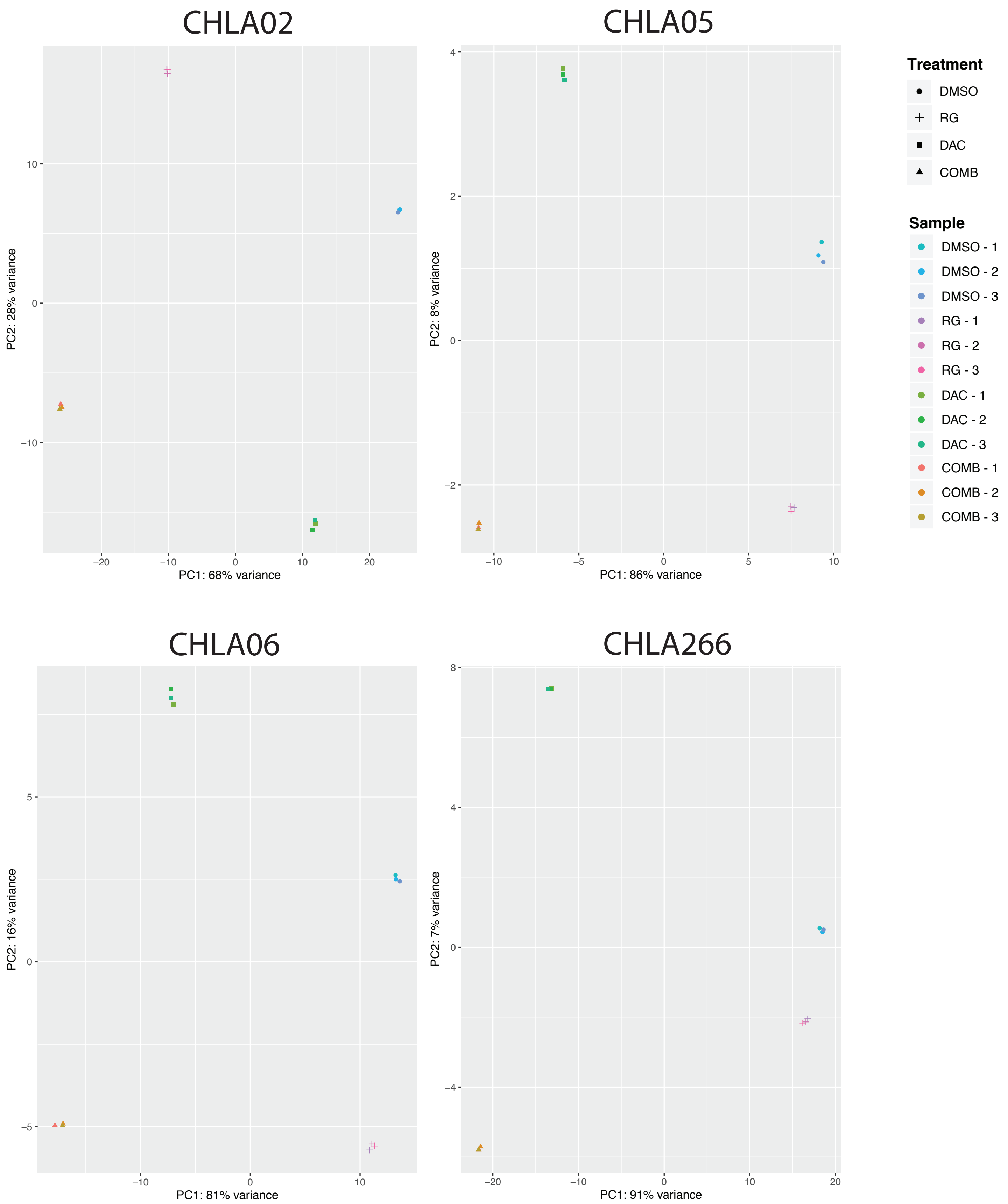
CHLA02
CHLA05
CHLA06
CHLA266
PC1: 68% variance
PC2: 28% variance
PC1: 86% variance
PC2: 8% variance
PC1: 81% variance
PC2: 16% variance
PC1: 91% variance
PC2: 7% variance
Treatment
DMSO
RG
DAC
COMB
Sample
DMSO - 1
DMSO - 2
DMSO - 3
RG - 1
RG - 2
RG - 3
DAC - 1
DAC - 2
DAC - 3
COMB - 1
COMB - 2
COMB - 3
