# Supplementary material for: Disordered DNA methylation leads to targetable transcriptional plasticity in ATRT
Source: Acta Neuropathol Commun. 2025 Dec 17;14:22. doi: 10.1186/s40478-025-02173-y (PMC12821819; doi:10.1186/s40478-025-02173-y)
Supplement: Supplementary file 6 — Supplementary Material 6. [file 40478_2025_2173_MOESM6_ESM.pdf]

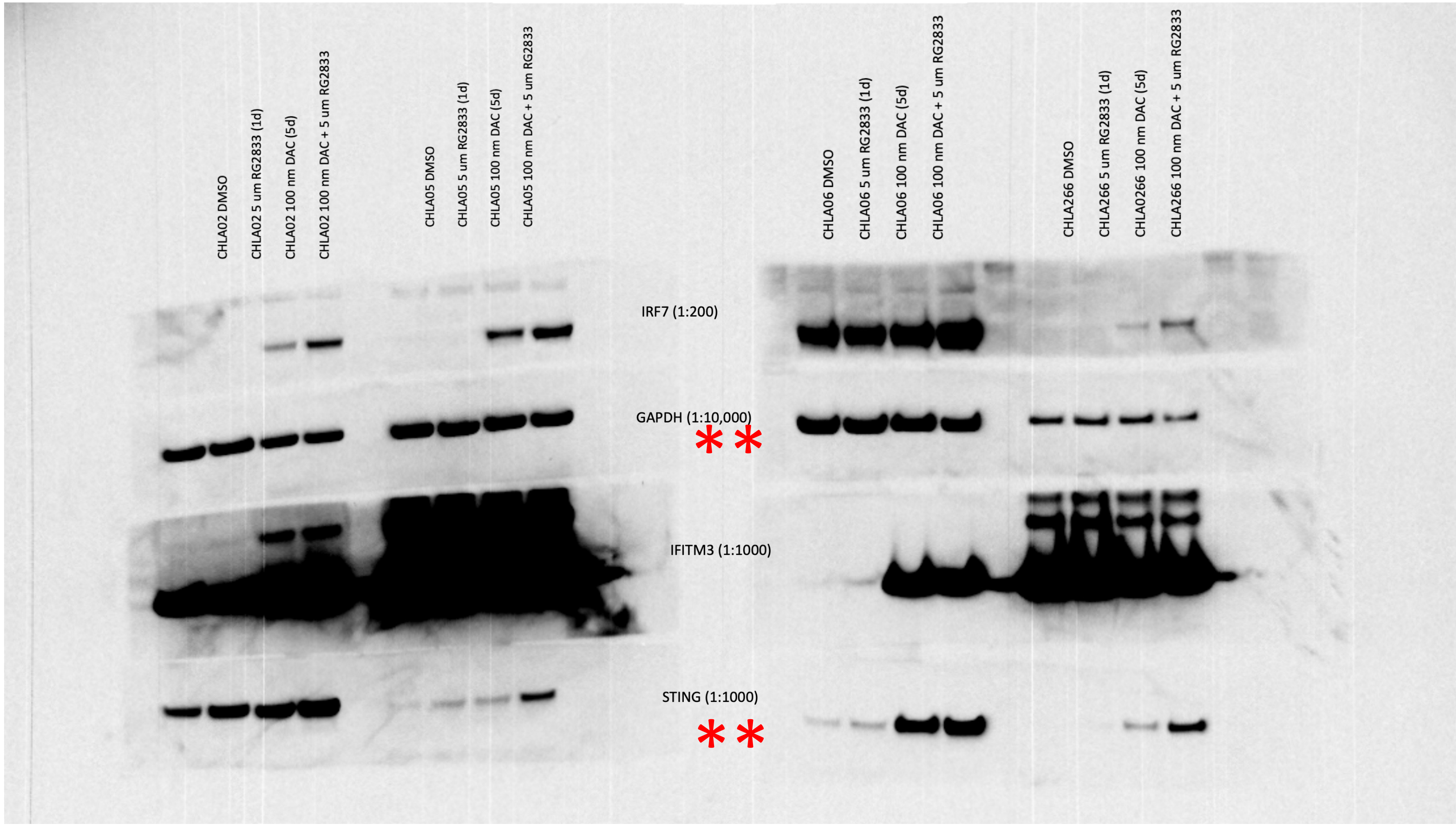

Supplementary file. Full uncropped Western blot images.

In all cases, blots were cut prior to application of primary antibody.

* Related to Figure 3

* Related to Figure 4

* Related to Figure S5

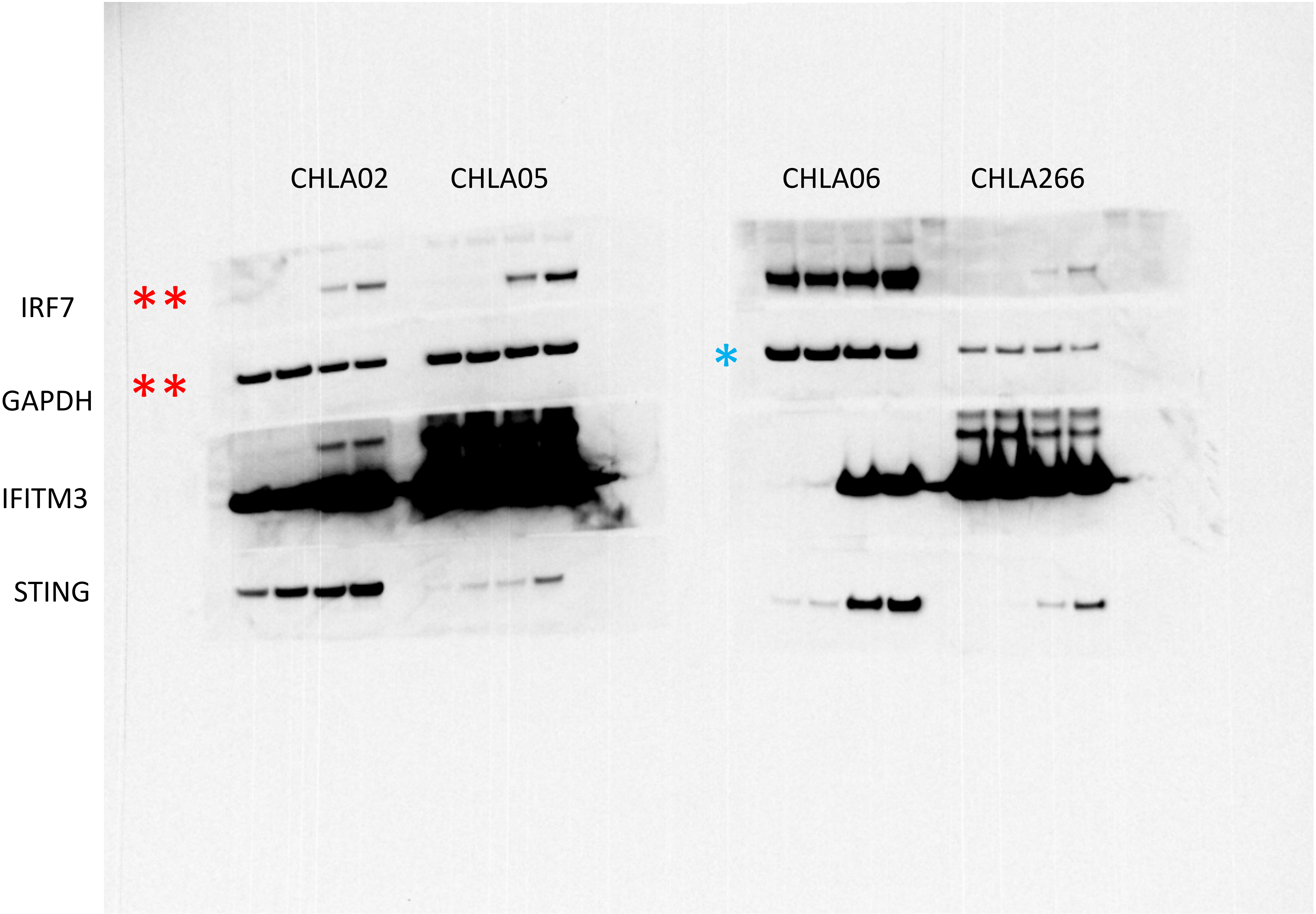
CHLA02
CHLA05
CHLA06
CHLA266
IRF7
GAPDH
IFITM3
STING
**
**
*

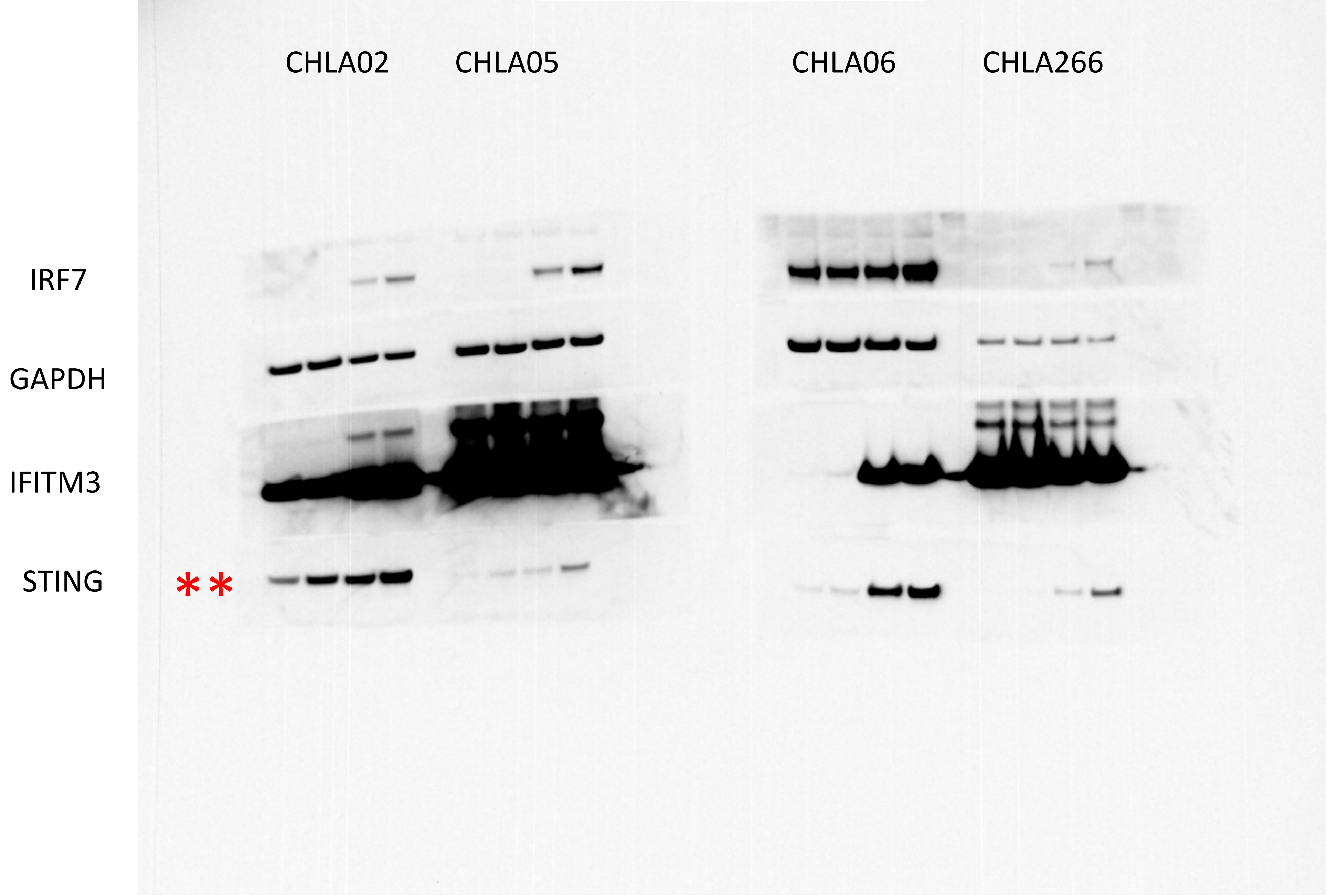
CHLA02
CHLA05
CHLA06
CHLA266
IRF7
GAPDH
IFITM3
STING
**

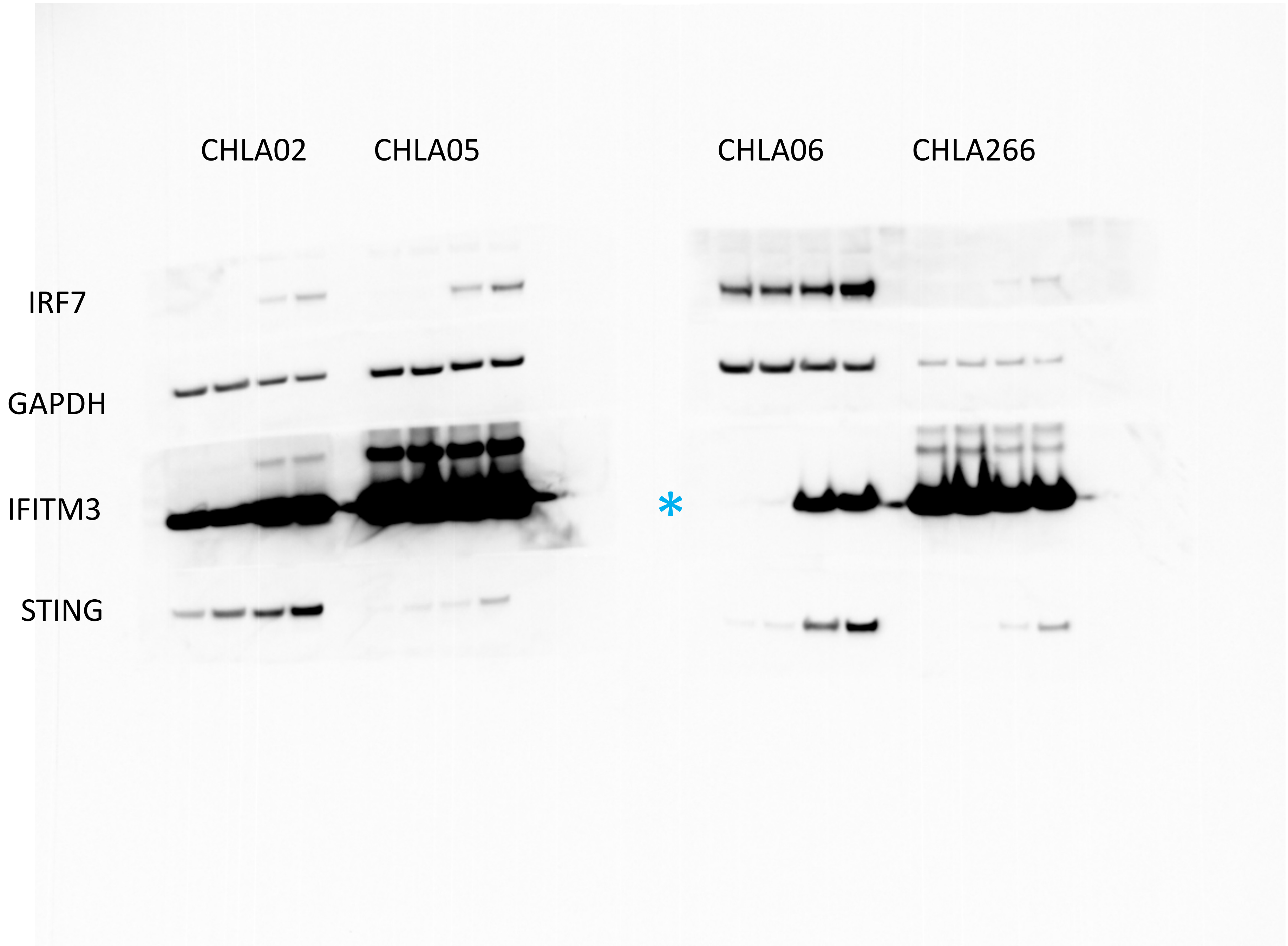
CHLA02
CHLA05
CHLA06
CHLA266
IRF7
GAPDH
IFITM3
*
STING

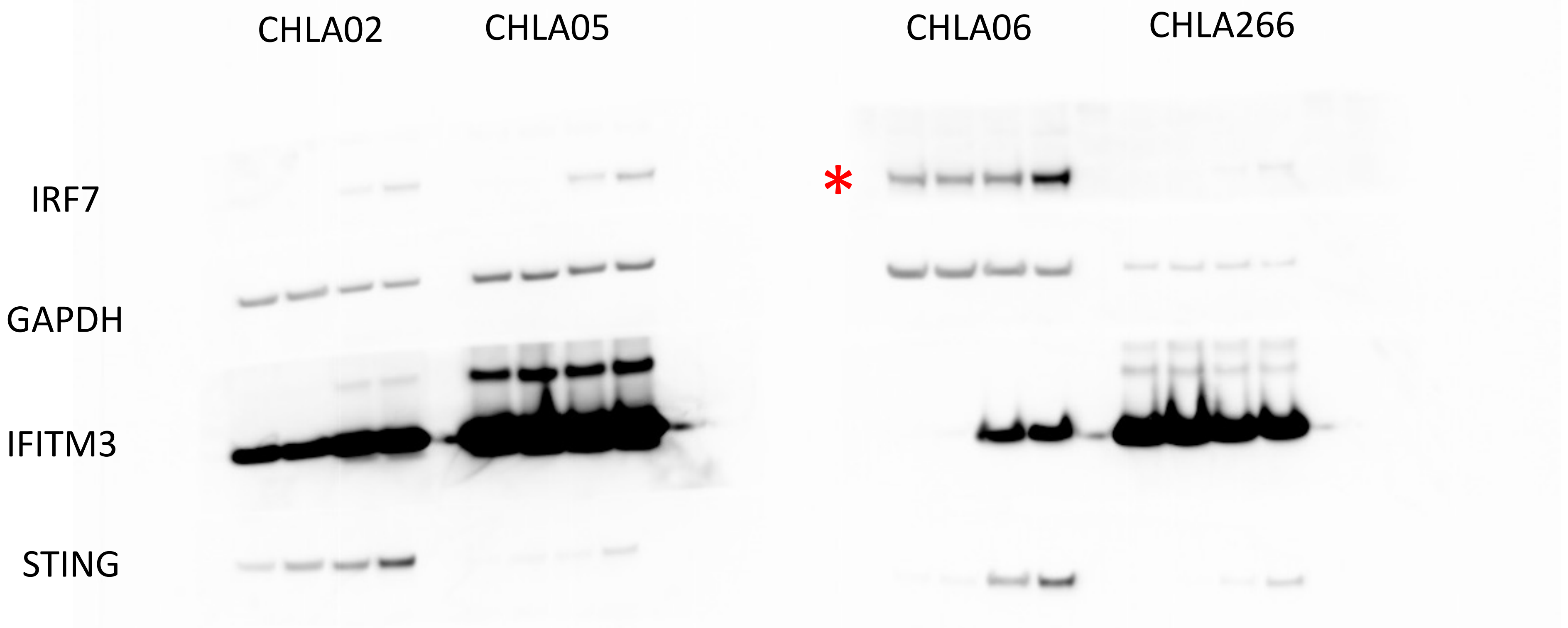
CHLA02
CHLA05
CHLA06
CHLA266
IRF7
*
GAPDH
IFITM3
STING

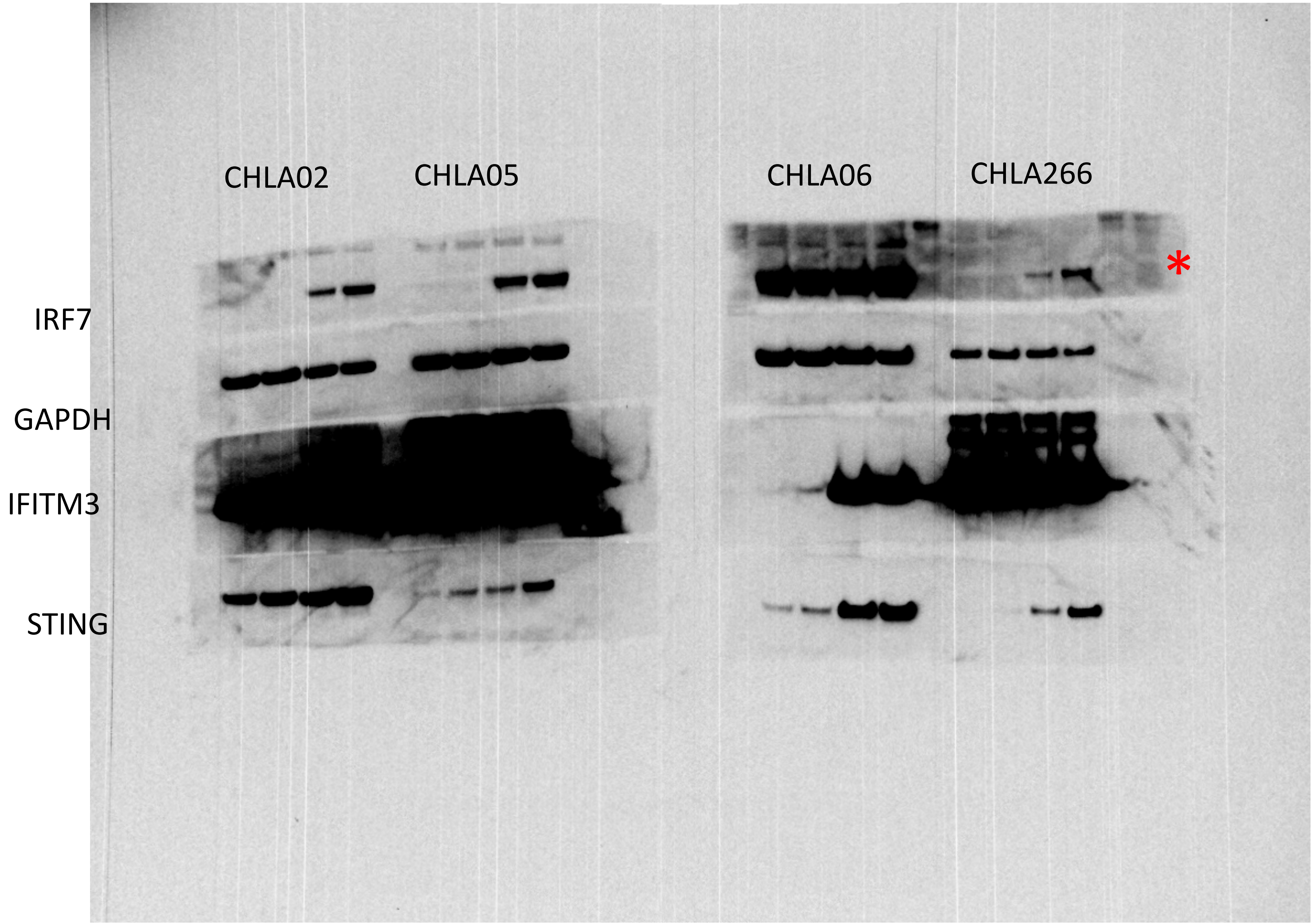
CHLA02
CHLA05
CHLA06
CHLA266
*
IRF7
GAPDH
IFITM3
STING

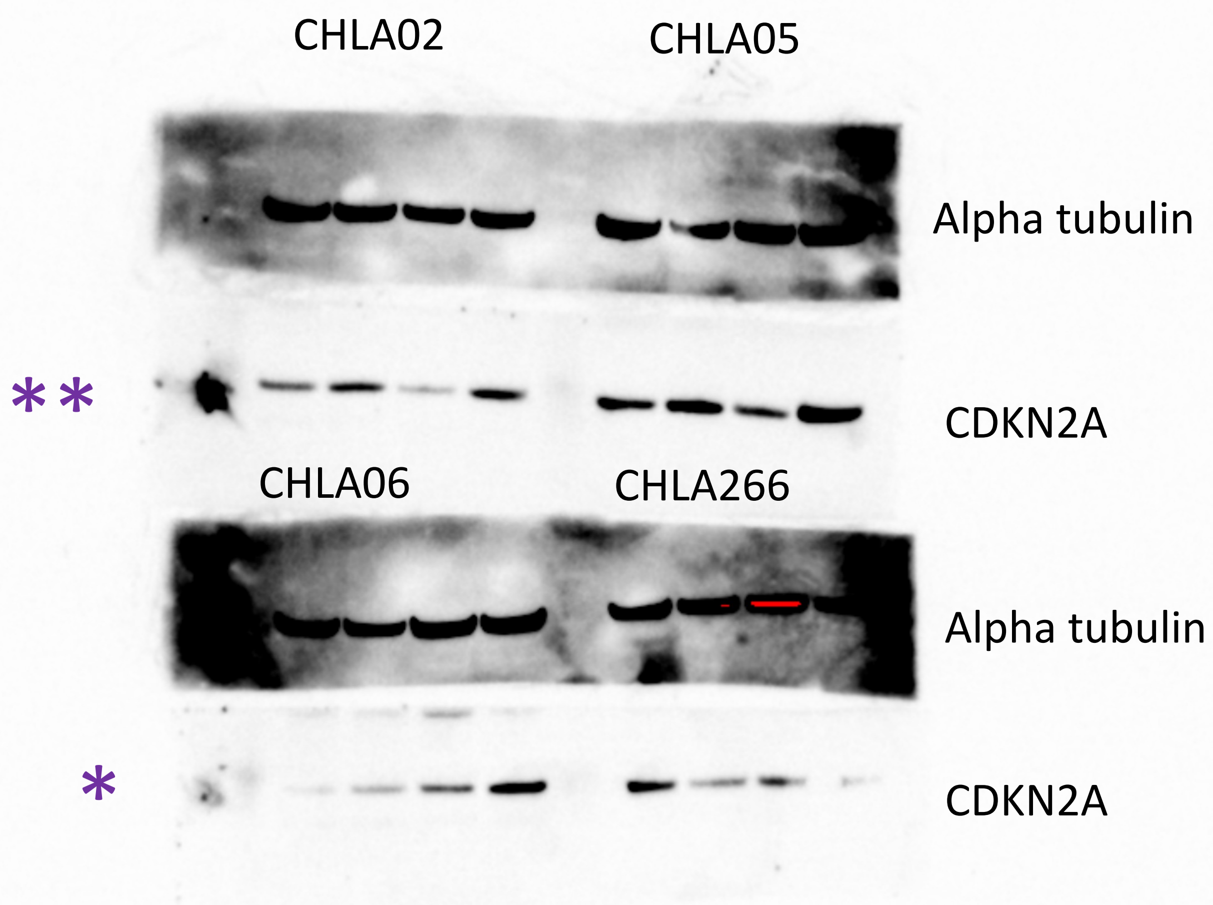
CHLA02
CHLA05
Alpha tubulin
**
CDKN2A
CHLA06
CHLA266
Alpha tubulin
*
CDKN2A

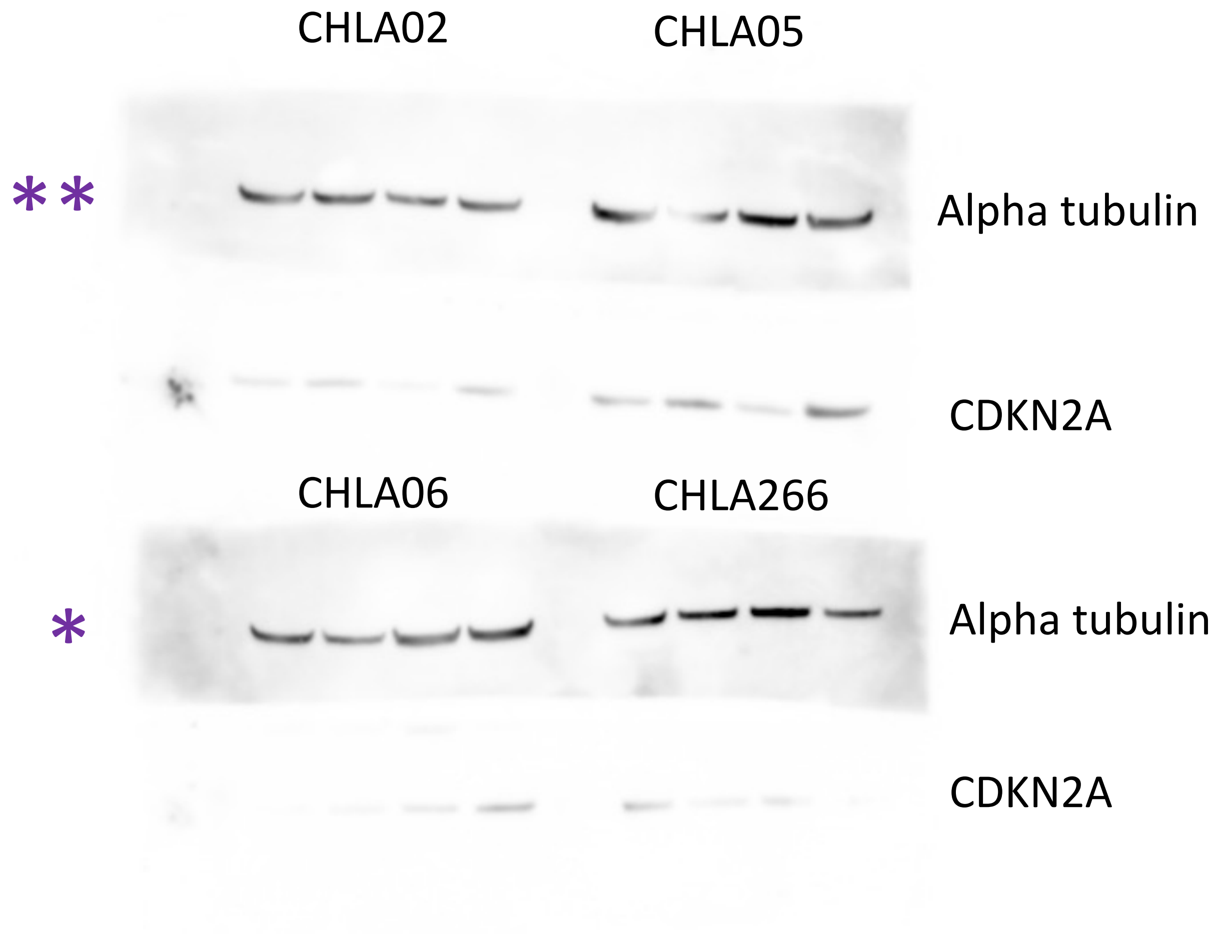
CHLA02
CHLA05
**
Alpha tubulin
CDKN2A
CHLA06
CHLA266
*
Alpha tubulin
CDKN2A

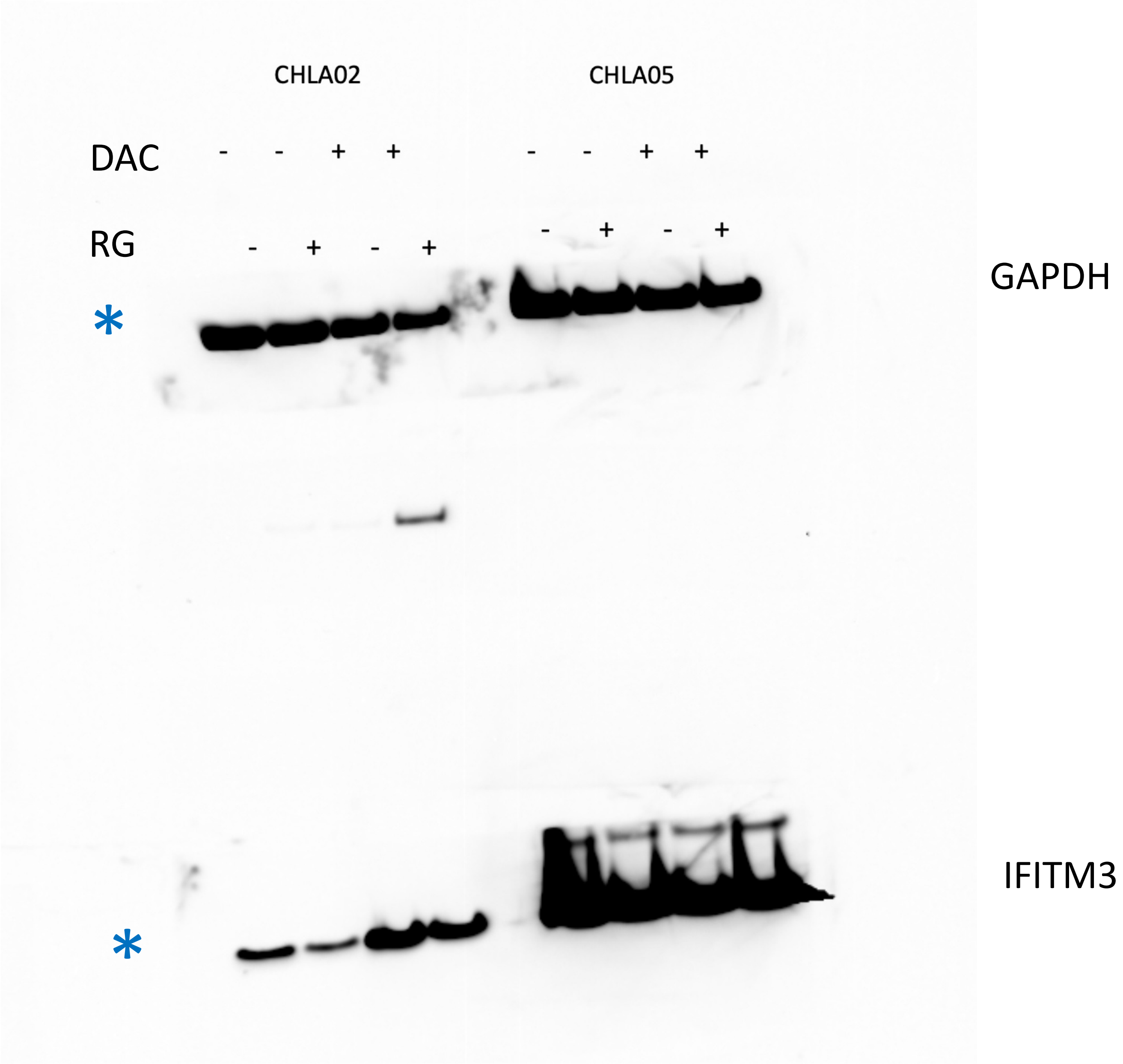
CHLA02
CHLA05
DAC - - + + - - + +
RG - + - + - + - +
GAPDH
IFITM3
*
*
