# Supplementary material for: Disordered DNA methylation leads to targetable transcriptional plasticity in ATRT
Source: Acta Neuropathol Commun. 2025 Dec 17;14:22. doi: 10.1186/s40478-025-02173-y (PMC12821819; doi:10.1186/s40478-025-02173-y)

CHLA02

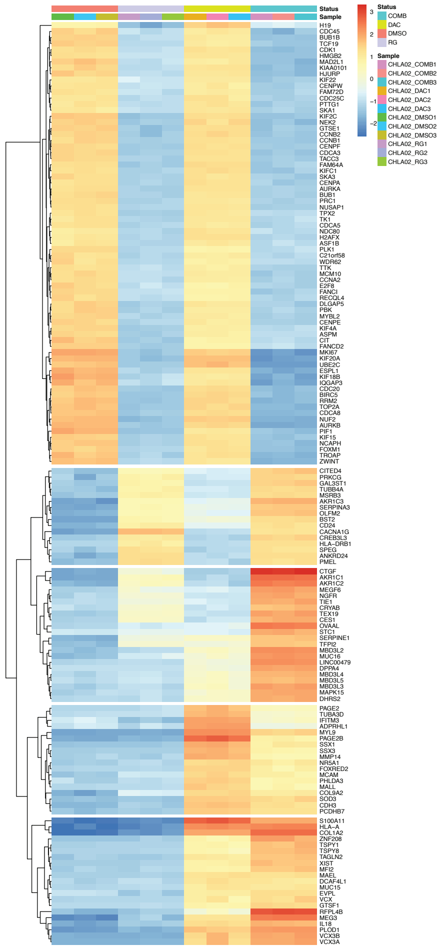

CHLA05

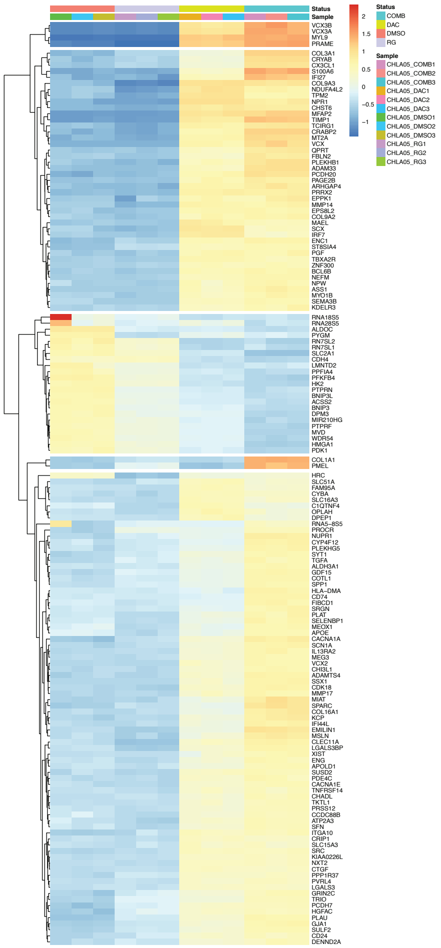

CHLA06

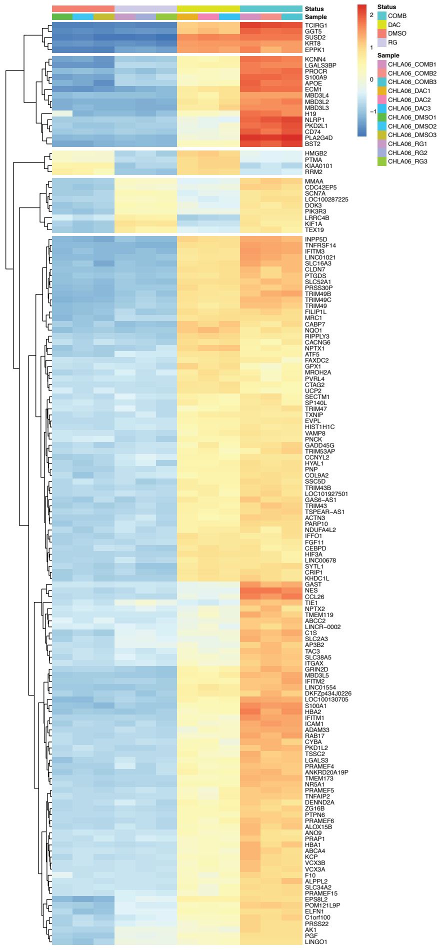

CHLA266

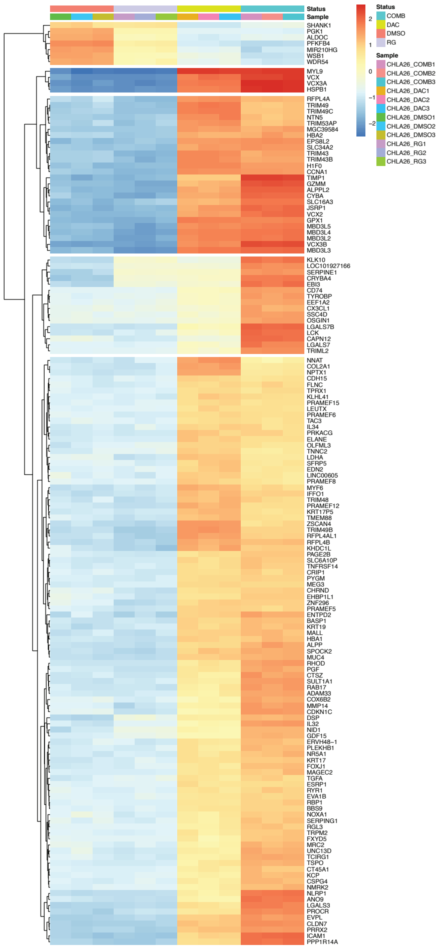

Supplement: Supplementary file 4 — Supplementary Material 4. Figure 4. Heatmaps of ATRT cell lines after treatment with DAC and RG2833. Heatmaps from figure 3b reshown with the gene names listed. Depicts the top 150 most differentially expressed genes in 4 ATRT cell lines: CHLA02, CHLA05, CHLA06, and CHLA266). Treatment order is as follows from left to right: DMSO, 5µM RG2833, 100 nM DAC, combination). All heat maps are divided into 5 clusters. Red, upregulated; blue, downregulated. [file 40478_2025_2173_MOESM4_ESM.pdf]
